# Inhibiting parasite proliferation using a rationally designed anti‐tubulin agent

**DOI:** 10.15252/emmm.202013818

**Published:** 2021-10-18

**Authors:** Natacha Gaillard, Ashwani Sharma, Izra Abbaali, Tianyang Liu, Fiona Shilliday, Alexander D Cook, Valentin Ehrhard, Mamata Bangera, Anthony J Roberts, Carolyn A Moores, Naomi Morrissette, Michel O Steinmetz

**Affiliations:** ^1^ Laboratory of Biomolecular Research Division of Biology and Chemistry Paul Scherrer Institut Villigen Switzerland; ^2^ Department of Molecular Biology and Biochemistry University of California Irvine California USA; ^3^ Institute of Structural and Molecular Biology Birkbeck, University of London London UK; ^4^ Biozentrum University of Basel Basel Switzerland

**Keywords:** anti‐parasite, microtubules, rational structure‐based drug design, species‐specific tubulin inhibitor, Microbiology, Virology & Host Pathogen Interaction, Structural Biology

## Abstract

Infectious diseases caused by apicomplexan parasites remain a global public health threat. The presence of multiple ligand‐binding sites in tubulin makes this protein an attractive target for anti‐parasite drug discovery. However, despite remarkable successes as anti‐cancer agents, the rational development of protozoan parasite‐specific tubulin drugs has been hindered by a lack of structural and biochemical information on protozoan tubulins. Here, we present atomic structures for a protozoan tubulin and microtubule and delineate the architectures of apicomplexan tubulin drug‐binding sites. Based on this information, we rationally designed the parasite‐specific tubulin inhibitor parabulin and show that it inhibits growth of parasites while displaying no effects on human cells. Our work presents for the first time the rational design of a species‐specific tubulin drug providing a framework to exploit structural differences between human and protozoa tubulin variants enabling the development of much‐needed, novel parasite inhibitors.

## Graphical abstract

In an effort to discover novel drug‐scaffolds targeting unique parasite proteins and pathways, specific inhibition of parasite tubulin was achieved using structure‐guided rational drug design.

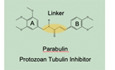

**The paper explained**

**Problem**

Protozoan parasites belonging to phylum Apicomplexa are responsible for several diseases including malaria, toxoplasmosis, and cryptosporidiosis. Drug administration remains the preferred treatment strategy for most of these diseases. However, a lack of safe and effective drugs coupled with the emergence of multiple drug‐resistant parasites necessitates the urgent discovery of novel drug scaffolds targeting unique parasite proteins and pathways.

**Results**

Inhibition of cell division by using tubulin targeting cytotoxic compounds has been a successful strategy for cancer treatment. Implementing a similar strategy to arrest parasite replication using protozoan tubulin‐specific inhibitors offers an attractive avenue toward anti‐apicomplexan drug discovery. However, this has been hampered by the lack of biochemical and structural knowledge on protozoan tubulins. In this work, we provide a structural description of apicomplexan tubulin drug‐binding sites together with the comparison to their mammalian counterparts and use this information to rationally design a parasite‐specific tubulin inhibitor dubbed “parabulin”. We show that parabulin specifically inhibits growth of protozoan parasites and inhibits their microtubule formation *in vitro*, while displaying no adverse effect on host cells or microtubules.

**Impact**

Our work presents for the first time the rational design of a species‐specific tubulin inhibitor, providing a framework to exploit structural differences between human and protozoa tubulin variants, which will enable the development of much‐needed, novel anti‐parasite drugs.

## Introduction

Apicomplexan parasites represent a large group of obligate intracellular pathogens that cause medically and agriculturally significant human and animal diseases including malaria, toxoplasmosis, babesiosis, cryptosporidiosis, and eimeriosis. The recent emergence of multiple drug‐resistant parasites is a serious threat to global health security and thus necessitates the urgent development of drugs targeting novel parasite proteins and pathways (Menard & Dondorp, 2017; Uwimana *et al*, 2020). Successful parasitism relies on the ability of parasites to rapidly invade host cells, to evade the host immune response, and to maintain robust growth within host cells. The parasite microtubule (MT) cytoskeleton plays a central role in the process of parasite replication and it also constitutes a key component of the parasite invasion machinery (reviewed in Morrissette (2015)).

MTs are assembled from αβ‐tubulin heterodimers and undergo phases of growth and shrinkage, a property termed dynamic instability (reviewed in Akhmanova and Steinmetz (2015)). MT‐targeting agents are among the most important drugs used in medical therapies to combat cancer. They act by either stabilizing or destabilizing MTs (Dumontet & Jordan, 2010). Recent advances in the structural characterization of tubulin– and MT–agent complexes have led to the identification of six distinct drug‐binding sites in mammalian tubulin (reviewed in Steinmetz and Prota (2018)). Although tubulin is highly conserved among eukaryotes, protozoan tubulins are much less sensitive to mammalian MT‐targeting agents such as colchicine (King & Beams, 1940; Bejon *et al*, 1997; Morrissette & Sibley, 2002). Similarly, dinitroaniline compounds disrupt specifically plant and protozoan parasite MTs but not mammalian MTs (Fennell *et al*, 2008). These observations imply that fine structural differences in the architecture of drug‐binding sites between protozoan and mammalian tubulin could be exploited to develop novel parasite‐specific anti‐tubulin drugs. Additionally, by targeting tubulin—an essential protein that contains multiple drug‐binding sites—combination therapies that engage distinct pockets at the same time could represent a unique strategy to circumvent the development of drug resistance.

## Results and Discussion

To analyze the entire spectrum of established, or any new putative additional, drug‐binding sites in apicomplexan tubulin, we need structural information on both the “straight”, assembled conformation of tubulin in MTs and the “curved” conformation of its free form in solution (Knossow *et al*, 2020). As attempts to produce recombinant apicomplexan tubulin similar to what has been achieved for human tubulin (Ti *et al*, 2016) have proved unsuccessful, we decided to isolate tubulin dimers from the free‐living ciliate *Tetrahymena thermophila*, as its tubulin is abundantly expressed and nearly identical (87–95% identity) to apicomplexan tubulins. Ciliates and apicomplexans both belong to the alveolate group (Dorrell *et al*, 2013), and their tubulins are sensitive to dinitroanilines (Lyons‐Abbott *et al*, 2010), suggesting that *T. thermophila* tubulin can be used as a proxy to study the interaction of ligands with apicomplexan tubulin.

**Figure 1:**
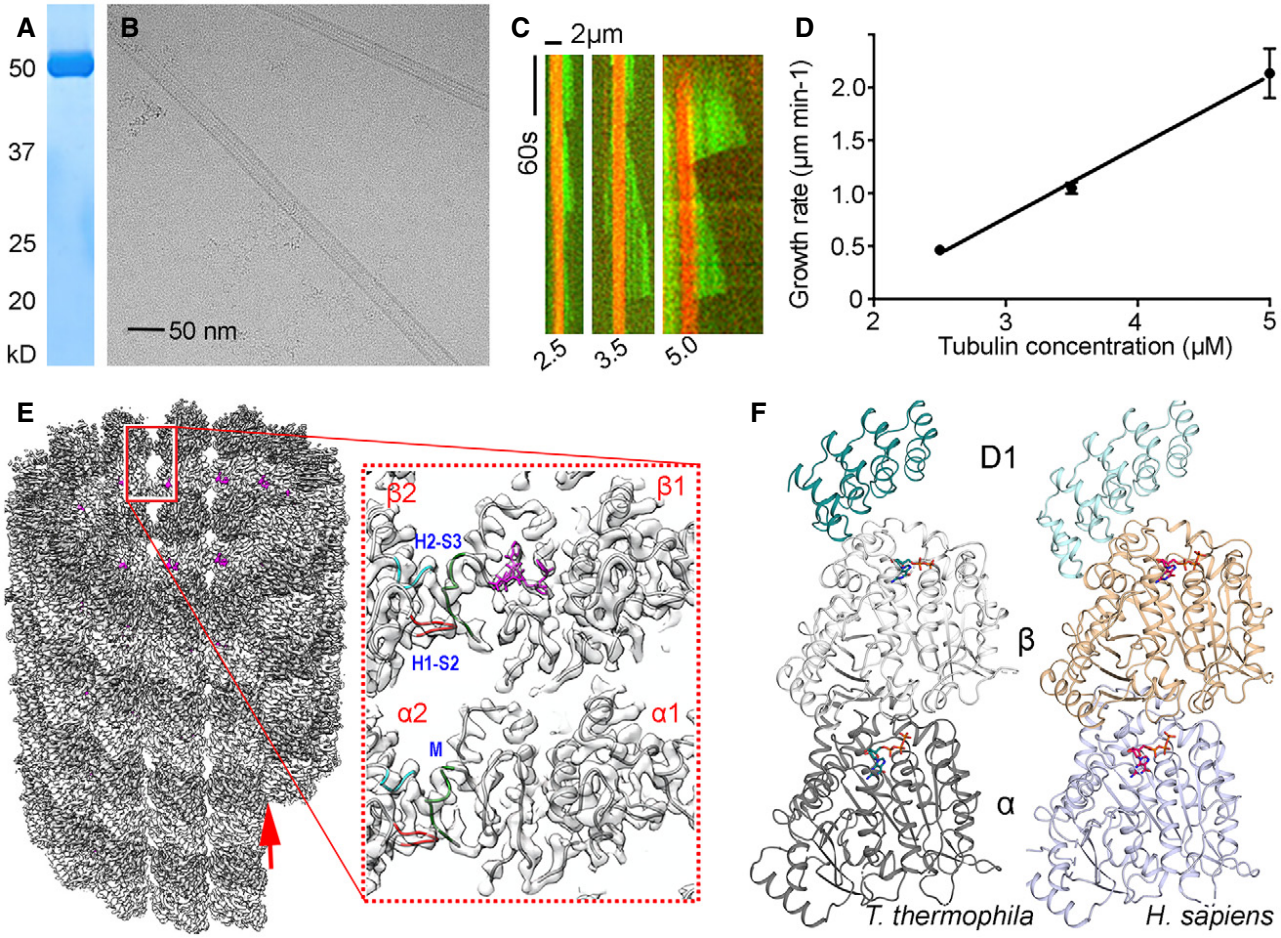
Protozoan and human tubulin and microtubule structures Coomassie‐stained SDS–PAGE showing purified *T. thermophila* tubulin.A representative cryo‐electron micrograph of paclitaxel‐stabilized *T. thermophila* MTs.Kymographs obtained by TIRF microscopy showing the dynamics of *T. thermophila* MTs at the indicated concentrations in µM.Plot of *T. thermophila* MT plus‐end growth rates as a function of tubulin concentration. Error bars represent the mean ± 95% CI. For each concentration, data are obtained from at least 2 independent experiments (*n* = 41).Cryo‐electron microscopy density map of a 14 protofilament, paclitaxel‐stabilized *T. thermophila* MT with pseudo‐helical symmetry applied. Left: outside view. The α‐ and β‐tubulin monomers are shown in surface representation and colored in dark and light gray, respectively. Red arrow shows the location of the seam. Right: tubulin dimer models fitted into a sliced, transparent and zoomed‐in view. The paclitaxel structure in stick representation is highlighted in magenta. Lateral contacts between protofilaments are indicated in blue (H2‐S3 in α2 and β2), red(H1‐S2 in α2 and β2), and green (M loop in α1 and β1).Cartoon representation of the crystal structures of *T. thermophila* tubulin‐D1 (left) and recombinant *H. sapiens t*ubulin‐D1 (right) complexes. Bound nucleotides are displayed in stick representation, the α‐ and β‐tubulin monomers in dark and light gray ribbons for *T. thermophila* tubulin and light blue and wheat ribbons for *H. sapiens t*ubulin, respectively.

Purified *T. thermophila* tubulin (Lyons‐Abbott *et al*, 2010) (Fig 1A) readily polymerized into MTs *in vitro* as revealed by cryo‐electron microscopy (Fig 1B). We used total internal reflection fluorescence (TIRF) microscopy to assess *T. thermophila* MT dynamics (Telley *et al*, 2011). When tested at a standard concentration range used to observe bovine brain MT dynamics (10–15 µM tubulin), many, long MTs filled the entire field of view (Appendix Fig S1). However, MT dynamics could readily be observed and quantified for *T. thermophila* tubulin at concentrations of less than 5 µM (which is below the critical concentration for bovine brain tubulin under these conditions, Fig 1C). *T. thermophila* MT growth rates (Fig 1D) were much faster compared to mammalian brain tubulin (growth rate of 2 μm‐min^−1^ at 5 μM for *T. thermophila* compared to 0.5 μm‐min^−1^ for *B*. *taurus*) but comparable to those observed with *C. elegans* tubulin (Chaaban *et al*, 2018).

To obtain structural information on *T. thermophila* tubulin and MTs, we employed X‐ray crystallography and cryo‐electron microscopy, respectively. We solved the structures of *T. thermophila* αβ‐tubulin in complex with DARPin1 (D1; (Pecqueur *et al*, 2012)) at 1.75 Å resolution (Fig 1F, Appendix Table S1) and paclitaxel‐stabilized *T. thermophila* MTs at 3.3 Å resolution (Fig 1E and Appendix Fig S2 and Table S2). To assess structural differences between protozoan and host mammalian tubulins, we also crystallized and solved the structure of recombinant human α1β3‐tubulin in complex with D1 at 1.9 Å resolution (Fig 1F and AppendixTable S1). The overall structure of *T. thermophila* tubulin is similar to that of human (RMSD of 0.49 Å over the 751 Cα atoms of curved αβ‐tubulin, and RMSD of 0.77 Å over the 801 Cα atoms of straight αβ‐tubulin) or yeast tubulin (Ayaz *et al*, 2014) (RMSD of 0.73 Å over the 755 Cα atoms of curved αβ‐tubulin, and RMSD of 0.796 Å over the 789 Cα atoms of straight αβ‐tubulin).

**Figure 2:**
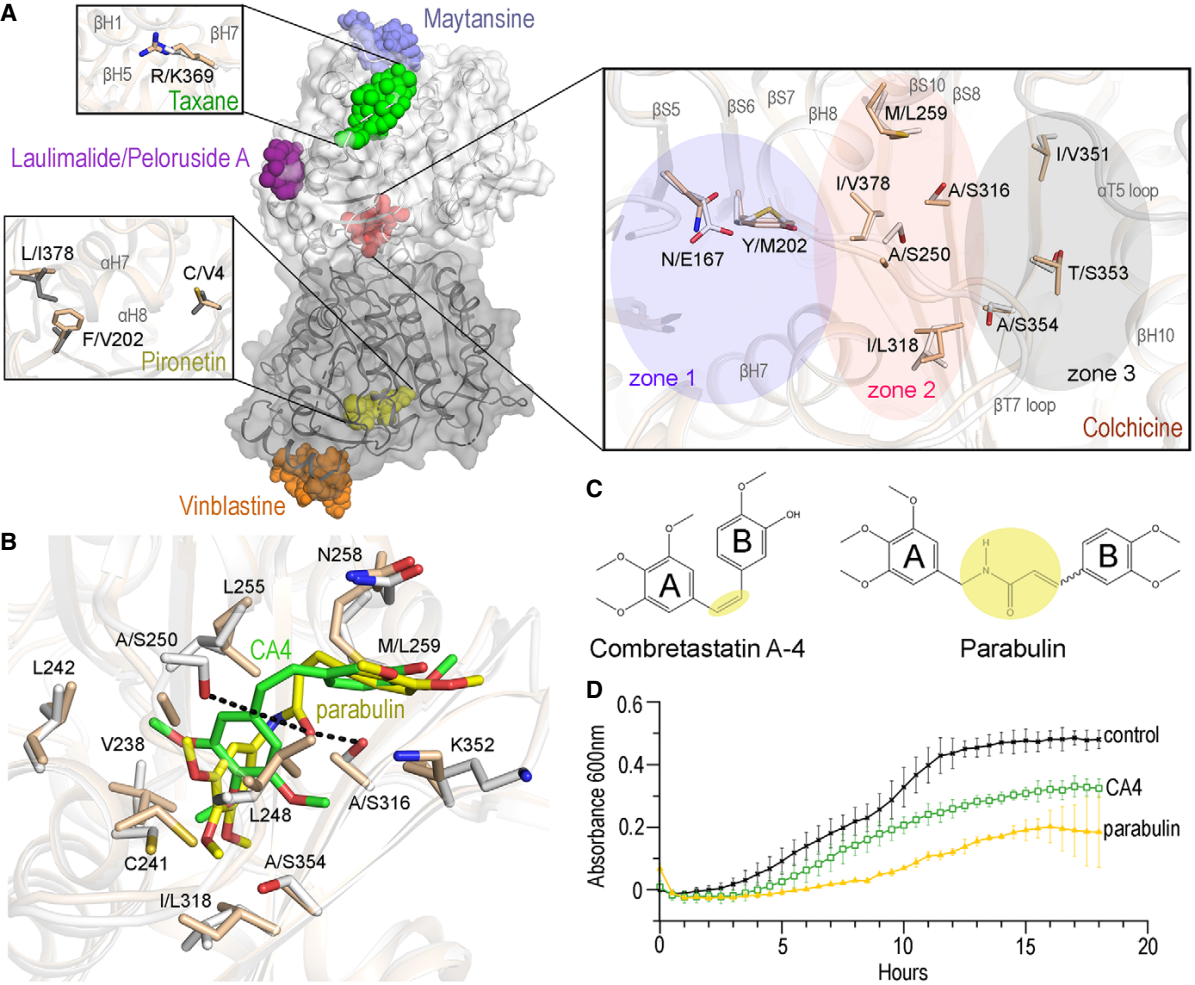
Analysis of protozoan–tubulin drug‐binding sites *T. thermophila* tubulin structure highlighting the six distinct drug‐binding sites found in mammalian tubulin. Enlargements show differences in the taxane, pironetin, and colchicine sites between *T. thermophila* (light grey) and *H. sapiens* (wheat) tubulin. The three distinct zones of the colchicine site are highlighted by colored transparent ovals. Amino acid residue differences between human and protozoa tubulin are indicated. Residues are displayed in stick representation and color‐coded accordingly to elements (N, blue; O, red; S, yellow; for human and protozoa, respectively).A zoom out of the colchicine site of *T. thermophila* tubulin showing the atomic arrangement of modeled CA4 (green) and parabulin (yellow).Chemical structures of CA4 and parabulin. The two CA4 rings, denoted A and B, are highlighted as well as the linker (yellow).Plot showing the time course of *T. thermophila* cell growth in the presence of CA4 (20 µM) or parabulin (50 µM; error bars corresponding to the standard deviation of triplicate biological measurements). Control experiments are done with DMSO alone.

**Figure EV1:**
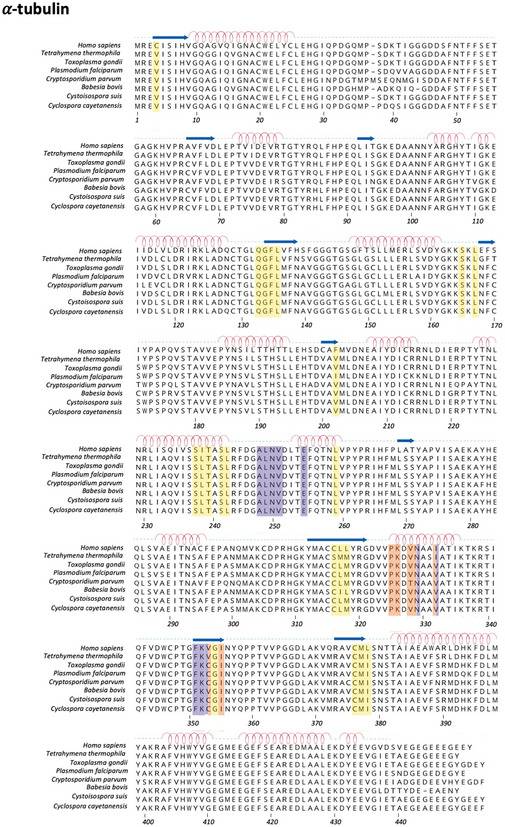
Multiple sequence alignment of α‐tubulin and mapping of known drug‐binding sites UniProtKB/Swiss‐Prot sequence accession identifiers are as follows: *Homo sapiens*, Hs_TBA1B; *Tetrahymena thermophila*, Tetrahymena_α‐Tubulin (P41351); *Toxoplasma gondii*, TGME49_316400; *Plasmodium falciparum*, PF3D7_0422300; *Cryptosporidium parvum*, cgd4_2860; *Babesia bovis*, BBOV_III002820; *Cystoisospora suis* CSUI_008696; *Cyclospora cayetanensis*, cyc_07446. Residues defining the following drug‐binding sites are highlighted: yellow, pironetin site; orange, vinblastine site; violet, eribulin site.

**Figure EV2:**
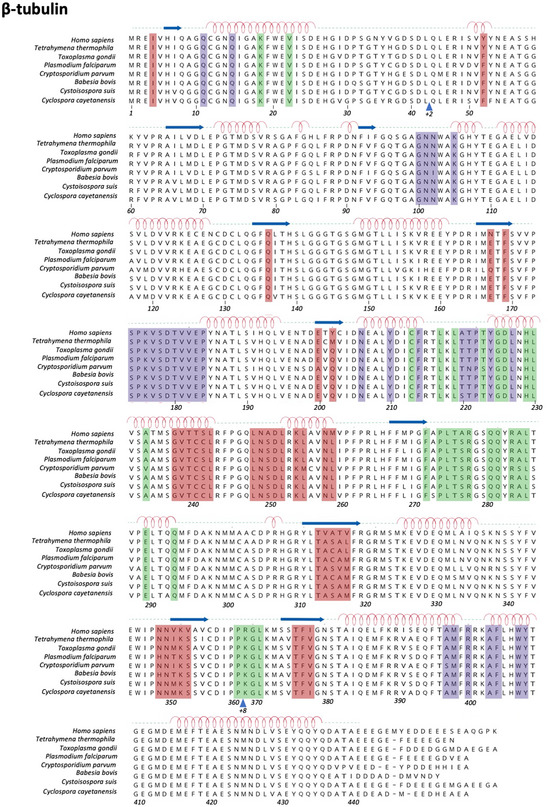
Multiple sequence alignment of β‐tubulin and mapping of known drug‐binding sites UniProtKB/Swiss‐Prot sequence accession identifiers are as follows: *Homo sapiens*, Hs_TBB3; *Tetrahymena thermophila*, Tetrahymena_BTUB1 (P41352); *Toxoplasma gondii*, TGME49_221620; *Plasmodium falciparum*, PF3D7_1008700; *Cryptosporidium parvum*, cgd6_4760; *Babesia bovis*, BBOV_III004850; *Cystoisospora suis*, CSUI_006169; *Cyclospora cayetanensis*, cyc_00127. Residues defining the following drug‐binding sites are highlighted: red, colchicine site; green, taxane site; violet, maytansine site.

**Figure EV3:**
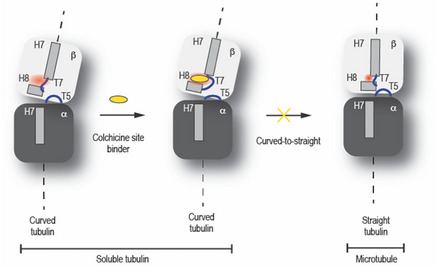
Structural comparison and mechanism of action of colchicine‐site ligands Schematic representation of the mechanism of action of colchicine‐site ligands. (1) Soluble tubulin displays a characteristic curved conformation. (2) colchicine‐site ligands (yellow sphere) block tubulin in the curved conformation by inhibiting the movement of helix βH7, βH8, βT7 loop, and ⍺T5 loop thus preventing MT formation (3).

**Figure EV4:**
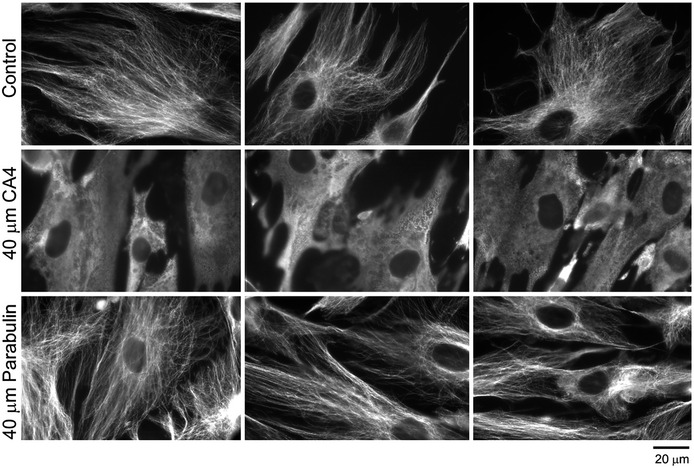
Activity of CA4 and parabulin on vertebrate cell MTs Tubulin immunofluorescence showing the effect of 40 μM CA4 (middle row) and 40 μM parabulin (bottom row) compared to a null control (top row) on cultured human fibroblast cells. Both null control and parabulin samples have intact cytoplasmic MT arrays, whereas CA4 treatment completely disrupts MTs, leading to a diffuse distribution of free tubulin dimers.

Next, we compared the details of all six known drug‐binding sites in mammalian tubulin (reviewed in (Steinmetz & Prota, 2018)) with the equivalent regions in *T. thermophila* tubulin to identify structurally unique features of protozoan tubulin (Fig 2A) using the three structures generated here as well as the published recombinant human MT structure (Vemu *et al*, 2016). The residue composition of the maytansine, laulimalide/peloruside, and vinblastine sites are identical between *T. thermophila* and human tubulin (Figs EV3 and EV4). In the case of the taxane and pironetin sites, we found differences in one and three amino acid positions, respectively (Figs 2A and EV1 and EV2). The most prominent differences between *T. thermophila* and human tubulin were observed for the colchicine site that is located at the α‐β intra‐tubulin dimer interface (Ravelli *et al*, 2004). It is formed by residues of helices H7 and H8, loop T7, and strands S8 and S9 of β‐tubulin and is completed by residues of loop T5 of α‐tubulin (Fig 2A). Tubulin dimers undergo a “curved‐to‐straight” conformational change upon incorporation into MTs, which requires rotational movements of strands S8 and S9 and a translation of helix H7 in both tubulin monomers and leads to a loss of the colchicine site (Ravelli *et al*, 2004; Wang *et al*, 2016; Sharma *et al*, 2017). Ligands that bind to the colchicine site block this conformational change and thus “freeze” tubulin in the MT incompatible, curved conformation (Fig EV3).

The structure of the colchicine site of mammalian tubulin is extensively characterized and contains three distinct zones: a central zone and two flanking accessory zones (Massarotti *et al*, 2012). We modeled the mammalian tubulin‐bound conformation of colchicine (Prota *et al*, 2014) in the context of the *T. thermophila* tubulin and compared it to human tubulin (see methods). Notably, the residues of human tubulin of the colchicine site among different human tubulin isoforms are overall largely conserved (AppendixFigs S3 and S4). However, as shown in Fig 2A and Fig EV2A, we observed amino acid differences in all the three zones of the *T. thermophila* colchicine site compared to all human isoforms. To assess whether such differences can affect the drug sensitivity at the organism level, we performed turbidity‐based *T. thermophila* growth‐inhibition assays in the presence of the three well‐characterized colchicine‐site ligands colchicine, combretastatin A4 (CA4), and plinabulin, all of which are known to bind at distinct zones of the colchicine site (Appendix Fig S5A). As shown in Appendix Fig S5B, CA4 showed a partial *T. thermophila* growth inhibition activity in between the ones of plinabulin (strongest effect) and colchicine (no effect). We decided to focus on CA4 as a starting point for optimization as we reasoned that it has the best chance to be further developed into an anti‐parasite‐specific agent. CA4 is a well‐known anti‐cancer drug currently in several phase 3 clinical trials (Grisham *et al*, 2018). It binds at zones 1 and 2 of mammalian tubulin with its 3,4,5‐methoxy‐substituted ring (ring A) making interactions with residues βV238, βS/C241, βL242, βA250 (serine in protozoa), βL255, βA316 (serine in protozoa), βI318 (leucine in protozoa), and βI378 (valine in protozoa) in zone 2 of the colchicine site, and its 3’‐hydroxy‐4’‐methoxy‐substituted ring (ring B) making interactions with the zone 1 residues βN258, βK352, βA316 (serine in protozoa), βM259 (leucine in protozoa), βN349, and αT179 of the colchicine‐site (Fig 2B) (Gaspari *et al*, 2017).

Based on structural modeling, we observed that rings A and B of CA4 could be similarly accommodated in the *T. thermophila* tubulin structure (Fig 2B). However, the aliphatic linker connecting the two rings of CA4, which is lined by hydrophobic amino acid side chains in human tubulin is replaced by polar residues in the case of protozoa tubulin (Fig 2A and B). Since a multiple sequence alignment indicates that βA250 and βA316 are conserved in all human β‐tubulin isoforms (Appendix Figs S3 and S4), we anticipated that the two key differences in the colchicine site of protozoan versus human tubulin (S/A250 and S/A316) would offer the opportunity to modify CA4 for parasite‐specific tubulin binding. To identify candidates that discriminate between protozoan and vertebrate tubulins, we assessed compounds from a commercially available ligand library (see methods). We searched for compounds that are similar to CA4 but which have alterations in the aliphatic linker connecting their two aromatic rings in CA4 to take advantage of the presence of the two colchicine‐site serine (or cysteine) residues at positions 250 and 316 in protozoan tubulin (Fig 2B and Appendix Fig S4), which engage this moiety of CA4. Molecules showing the most favorable binding poses, as judged by molecular modeling and structural analysis of the compounds in the *T. thermophila* tubulin crystal structure, were selected for *in vivo* testing (see Fig 2B for an example and supplemental methods).

**Figure 3:**
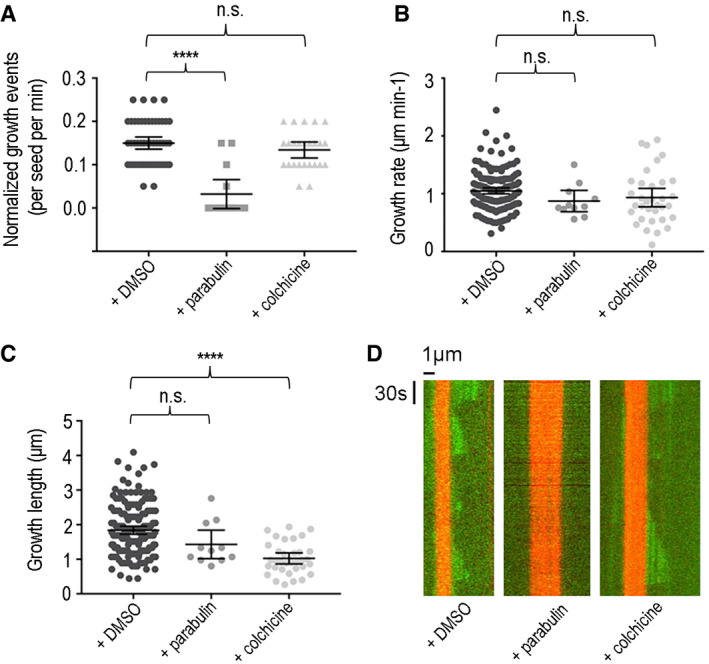
Parabulin inhibits Tt‐MT growth *in vitro* Plot showing quantification of the *T. thermophila* MT growth events in TIRF microscopy MT dynamics reconstitution assays in the presence of the indicated drugs. Error bars represent the mean ± 95% CI. For each concentration, data are obtained from at least 2 independent experiments. For growth rates and growth lengths, *n* = 41 (DMSO), *n* = 4 (0.04 μM parabulin), and *n* = 34 (0.04 μM colchicine). Rates were compared using one‐way ANOVA with Tukey’s multiple comparison test; DMSO vs parabulin *P* < 0001 (****); DMSO vs colchicine *P* = 0.40 (n.s., not significant).
*T. thermophila* MT growth rates in the presence of the indicated drugs (40 μM). Error bars represent the mean ± 95% CI (*n* = 4). Rates were compared using one‐way ANOVA with Tukey’s multiple comparison test; DMSO vs parabulin *P* = 0.25; DMSO vs colchicine *P* = 0.20 (n.s., not significant).
*T. thermophila* steady‐state MT length in the presence of the indicated drugs (40 μM). Error bars represent the mean ± 95% CI. For each concentration, data are obtained from at least 2 independent experiments. For growth rates and growth lengths, *n* = 41 (DMSO), *n* = 4 (0.04 μM parabulin), and *n* = 34 (0.04 μM colchicine). Rates were compared using one‐way ANOVA with Tukey’s multiple comparison test; DMSO vs parabulin *P* = 0.15 (n.s., not significant); DMSO vs colchicine *P* < 0001 (****).Representative kymographs obtained by TIRF microscopy showing the effect of parabulin and colchicine on dynamic *Tt‐*MTs.

Among the various compounds tested, only one compound—which we dubbed parabulin—inhibited *T. thermophila* cell growth (Fig 2C and D). To test whether parabulin can bind to tubulin, we assessed its activity *in vitro* on dynamic MTs using our TIRF microscopy reconstitution assay. In contrast to the dynamic periods of growth and shrinkage observed for *T. thermophila* MTs (Fig 1C), the addition of parabulin led to a striking reduction in the numbers of growth events (Fig 3A). The average number of growth events per MT seed decreased from 0.15 ± 0.001 to 0.03 ± 0.03 per min. When rare growth events occurred, no significant differences in growth rate (control vs parabulin *P*‐value = 0.25) or growth length (control vs parabulin *P*‐value = 0.15) were observed (Fig 3B–D). In control experiment, addition of colchicine had little effect on the dynamic properties of *T. thermophila* MTs (Fig 3A–D), while parabulin had no detectable effect on the dynamic properties of porcine brain MTs (Appendix Fig S1D). These data suggest that parabulin would sequester protozoan tubulin in a polymerization incompetent state by binding to its colchicine site and by preventing conformational changes required for microtubule formation. The few growth events that do take place in the presence of parabulin would therefore represent the residual unbound polymerization competent tubulin as indicated by similar to native microtubule dynamic properties. Together with our structural analyses, these results suggest that parabulin is a protozoan‐targeting MT‐destabilizing agent.

**Figure 4:**
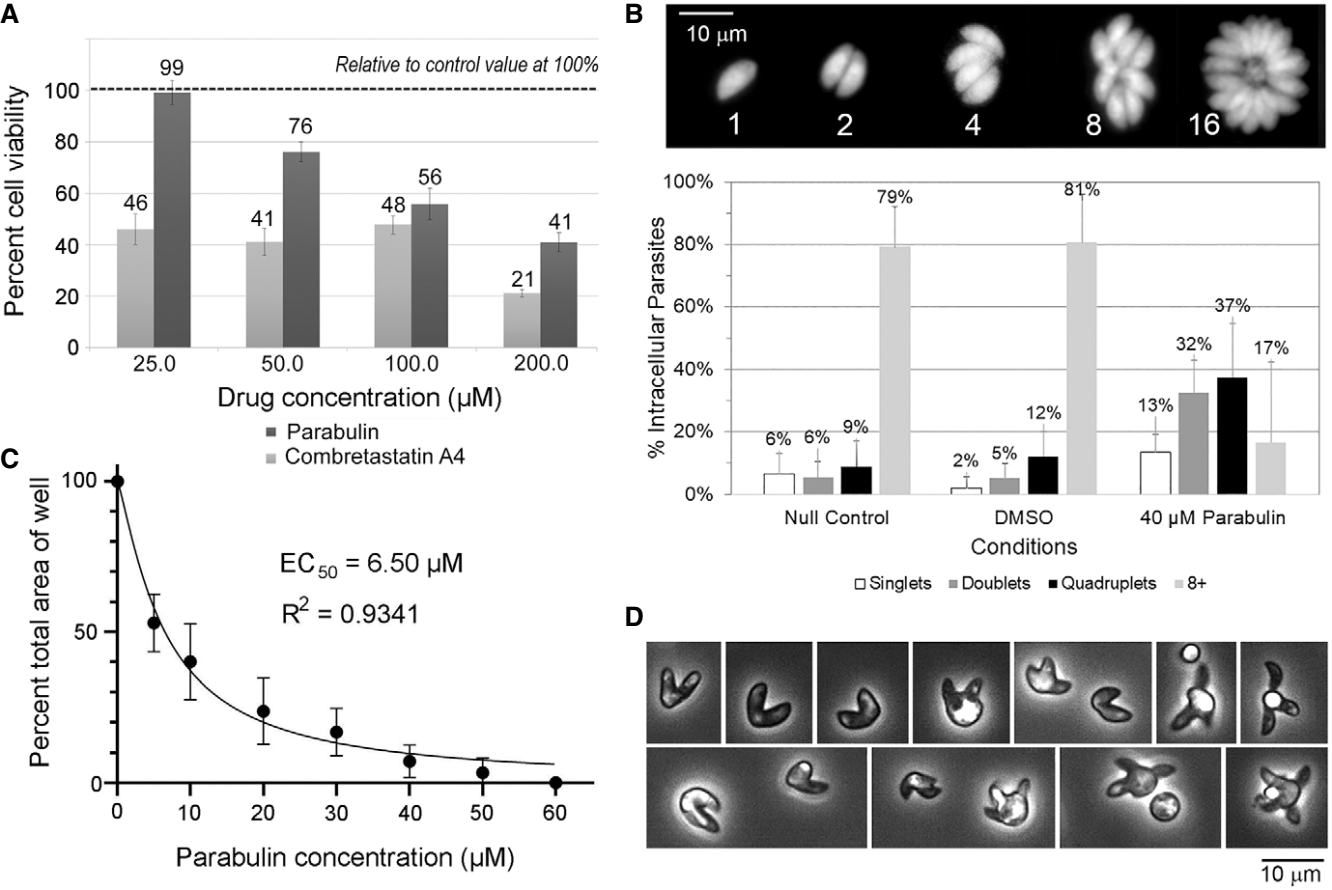
Parabulin inhibits parasite invasion and replication Evaluation of vertebrate cell toxicity using the MTT assay which detects metabolic activity proportionate to the number of viable cells in a sample. The absorbance measurements for all treatment conditions were normalized to the null control value which is set to 100% growth. Subconfluent human fibroblasts were used to visualize potential compound effects during proliferation. The results represent the average of 9 readings (3 biological replicates with 3 technical replicates of each treatment) ± standard error of the mean.Replication of GFP‐expressing *T. gondii* tachyzoites in individual intracellular vacuoles can be followed by live cell imaging. Since vacuoles containing more than eight tachyzoites are not always easy to score, we tallied vacuoles above eight as being 8+ in size. At 36 h, both null and vehicle control samples show most vacuoles harbor eight or more tachyzoites (three or more doublings). Vacuoles in parabulin‐treated cultures contain significantly fewer parasites, indicating that replication is slowed. The graph shows three biological replicates with values normalized to 100% for each condition and each replicate, error bars represent standard error of the mean between experiments.The parabulin EC_50_ value for *T. gondii* growth was determined using a plaque assay to measure a reduction in plaque area relative to control conditions. Error bars represent standard error of the mean. The results represent the average of 18 readings (6 biological replicates with 3 technical replicates of each treatment) ± standard error of the mean.Phase contrast of extracellular parasite cultures. Serial passage of *T. gondii* in 40–80 μM parabulin induces accumulation of aberrant tachyzoite forms. These arise with MT defects that disconnect coordinated nuclear division and daughter cell budding.

To assess the potential of parabulin as a bona fide anti‐parasitic agent, we evaluated its effects on cultures of human cells and growth of the apicomplexan parasite *Toxoplasma gondii* within human cells. When subconfluent (proliferating) fibroblasts were exposed to equivalent concentrations of parabulin or CA4 for 72 h, parabulin exhibited considerably lower cytotoxicity than CA4 (Fig 4A). CA4 has well‐defined anti‐mitotic activity due to its disruption of vertebrate MTs (Mooney *et al*, 2009). In contrast, parabulin had little to no effect on fibroblast growth or survival, indicating that it does not harbor significant cytotoxic activity (Appendix Fig S6A).

Immunofluorescence staining of tubulin in fibroblast monolayers treated with CA4 or parabulin indicates that unlike CA4, parabulin does not disrupt the MT cytoskeleton of fibroblasts (Fig EV4). To evaluate the effect of parabulin on parasite replication, GFP‐expressing *Toxoplasma* tachyzoites were added to fibroblast monolayers to allow invasion. After addition of parabulin, there was a significant reduction in parasite replication compared to control cultures (Fig 4B). Whereas most parasite vacuoles in control samples contained 8 or more parasites, meaning that these tachyzoites have doubled at least 3 times after invasion (Fig 4B, top), parabulin‐treated samples display vacuoles harboring significantly fewer parasites, indicating that replication is delayed by 1–3 replication cycles. We separately assessed whether parabulin treatment influences host cell invasion (infectivity) using a “red‐green/in‐out” assay that distinguishes extracellular and intracellular parasites (Huynh *et al*, 2003) and found that parabulin also reduces tachyzoite invasion of host cells (Appendix Fig S6B). We used a *T. gondii* plaque assay to determine the concentration at which parasite proliferation is reduced by half (EC_50_ value); this analysis captures effects on both invasion and intracellular replication because plaques arise after several rounds of invasion, replication, and parasite egress (Ma *et al*, 2008). When confluent fibroblast monolayers were infected with tachyzoites and left to grow for seven days, lytic parasite growth created visible holes in the vertebrate fibroblast monolayer. Both the plaque numbers and sizes were reduced by parabulin treatment. Quantification of the plaque area relative to control samples indicates that the parabulin has an EC_50_ value of 6.5 µM (Fig 4C). Although parasites do not display visible MT defects after short‐term treatment, when tachyzoites are serially passaged in parabulin, aberrant progenies accumulate (Fig 4D). These abnormal forms arise when nuclear division and daughter cell budding (cytokinesis) are disconnected, such as in the context of, for example, mutating tubulin (Ma *et al*, 2007). Taken together, these results substantiate our drug design strategy and reveal that parabulin is a specific inhibitor of protozoan MT functions.

Many of the current strategies to develop new therapeutic drugs to treat parasite infections focus on phenotypic screens to identify novel anti‐parasitic agents (Fernández *et al*, 2019). However, since parasites like *Plasmodium* develop resistance quickly after introduction of new therapies (Haldar *et al*, 2018), targeted strategies are also required to tackle deadly parasitic infections like malaria. Inhibiting essential parasite pathways using drug formulations targeting multiple sites on a single, essential parasite protein represents an attractive approach to controlling these parasitic infections. Here, we show that it is possible to specifically target parasite tubulin without harming the human host cell. We identified a compound that specifically alters parasite MTs and inhibits parasite replication and host cell invasion. Parabulin, which inhibits growth of protozoan MTs, complements the recent identification of a tubulin‐binding compound that selectively promotes growth of *Leishmania* and *Trypanosoma* (kinetoplastid) MTs to specifically inhibit parasite replication (Ullah *et al*, 2020). By pairing structural and biochemical studies with drug design and *Toxoplasma* assays, we confirm the potential of rationally developed MT‐targeting agents as anti‐parasitic treatments. Moreover, our structural information offers a unique basis to develop additional selective ligands that bind to distinct sites on parasite tubulins.

## Materials and Methods

### *Tetrahymena thermophila* culture and tubulin purification

*Tetrahymena thermophila* cells (strain SB210, Cornell University, USA) were cultured for 30 h under agitation at 30°C in 1 l SPP media (Sequestrene Proteose Peptone: 1% protease peptone, 0.1% yeast extract, 0.2% glucose, 33 μM FeCl_3_, and 1% Penicillin/Streptomycin/Amphotericin B Mix from PAN Biotech). Cells were collected by centrifugation for 15 min at 6,000 *g*, flash–frozen, and stored at −80°C.

Each tubulin purification used 48 to 72 l of cells. The cell pellets were resuspended in a final total volume of 250 ml of PME buffer (0.1 M PIPES pH 6.9, 1 mM MgCl_2_, 1 mM EGTA) plus protease inhibitor (0.5 mM phenylmethylsulfonyl fluoride and cOmplete^TM^ Roche), and sonicated on ice for 30 min (2 s ON, 2 s OFF, 30% amplitude macrotip VibraCell). The sonicated cells were cooled on ice for an additional 30 min before being spun down at 20,000 *g* for 40 min at 4°C. The resulting clarified supernatant was filtered through a 0.45‐micron filter then loaded onto a 5‐ml HiTrap DEAE Sepharose FF (GE Healthcare). The column was washed with 2 volumes of PME buffer followed by 4 volumes of PME buffer containing 100 mM KCl and 0.25 M glutamate. Bound tubulin was eluted with 2 volumes PME buffer containing 300 mM KCl and 0.75 M glutamate. An assembly‐competent tubulin fraction was purified by a cycle of polymerization–depolymerization: After elution from the DEAE column, 10 mM MgCl_2_, 2 mM GTP, and 8% DMSO (final concentration) were added to the pooled tubulin which was incubated at 37°C for 1 h. After spinning at 50,000 *g* for 30 min at 37°C, the supernatant was removed, and the polymerized tubulin pellet was resuspended in 100 μl cold PME buffer. The tube was incubated on ice for 15 min and then transferred into ice‐cold water for sonication 1 min 5 s ON 2 s OFF 30% amplitude with a macrotip (VibraCell). The tubulin was spun down once more at 50,000 *g* for 20 min at 4°C. The resulting supernatant consisted of pure *Tt*Tubulin which was used for crystallization and other biochemical assays.

### Labeling of tubulin for TIRF experiments

We added 5 μl of 1 M MgCl_2_, 20 μl of 100 mM GTP, and 1 ml glycerol to 40 mg of purified *Tt*Tubulin (2 ml volume) and incubated it for 1 h at 37°C. Polymerized tubulin was layered on top of 1 ml of warm (37°C) High pH Cushion (0.1 M NaHEPES, pH 8.6, 1 mM MgCl_2_, 1 mM EGTA, 60% (v/v) glycerol) and spun at 70,000 *g* for 20 min at 35°C. The supernatant above the cushion was aspirated and the supernatant–cushion interface rinsed twice with warm labeling buffer (0.1 M NaHEPES, pH 8.6, 1 mM MgCl_2_, 1 mM EGTA, 40% (v/v) glycerol). The pellet was resuspended in 500 μl of warm labeling buffer. Alexa Fluor^®^ 488 Carboxylic Acid was then added to the mixture in a 10‐fold molar excess. The labeling reaction was incubated for 10 min at 37°C before addition of 500 μl of Quench (2× BRB80, 100 mM K‐Glutamate, 40% (v/v) glycerol) with a further 5‐min incubation. In order to remove free dye, the quenched labeling reaction was layered onto 1 ml of Low pH Cushion (60% (v/v) glycerol in 1× BRB80) and spun at 70,000 *g* for 20 min at 35°C. The labeled tubulin pellet was resuspended in 400 μl of ice‐cold 1× BRB80 and incubated on ice for 30 min. Depolymerized labeled tubulin was spun at 70,000 *g* for 10 min at 4°C. The active fraction of 488‐*Tt* Tubulin was recovered by an additional cycle of polymerization–depolymerization. We added 4 mM MgCl_2_, 1 mM GTP, and half the volume of glycerol to the cold spin supernatant and incubated it for 30 min at 37°C. The polymerization reaction was layered on 1 mM Low pH Cushion, and the microtubules spun down at 70,000 *g* for 20 min at 37°C. The supernatant was aspirated, and the pellet washed with 1 ml of warm 1× BRB80 to remove any residual glycerol. The pellet was resuspended in 100 μl ice‐cold 1× BRB80 and incubated on ice for 30 min. A final spin at 70,000 *g* for 10 min at 4°C removed any aggregated tubulin. At this time, the supernatant was recovered, the tubulin concentration determined and the 488‐*Tt*Tubulin was concentrated to 3 mg/ml, aliquoted in 5 μl aliquots, and flash‐frozen in liquid nitrogen.

### Crystallization and structure solution

Darpin 1 was produced as previously described (Sharma *et al*, 2016). *Hs*T_βIII_D1 was crystallized as described previously (La Sala *et al*, 2019). The *Tt*TD1 complex was obtained by diluting pure tubulin to 1 mg/ml in water and 1 mM GTP with subtilisin (Sigma) added to a 1/100 weight ratio. After a 45‐min incubation at 25°C, the reaction was stopped with PMSF (2 mM final concentration) and MgCl_2_ was added to a final concentration of 1 mM. After 40 min on ice, the mixture was spun down at 70,000 *g* for 30 min at 4°C. Darpin 1 was added in a 1:1 molar ratio, and the complex was concentrated to 40 mg/ml. Crystals grew overnight in hanging drop vapor diffusion at 20°C in 0.2 M sodium sulfate, 10% DMSO, and 18% PEG 3350.

X‐ray diffraction data were collected at the PXIII beamline of the Swiss light source. Data were processed using XDS software package (Kabsch, 2010a, 2010b). *Hs*T_βIII_D1 crystallized in the space group P12_1_1 with a single molecule in the asymmetric unit. Structure solution was performed by the molecular replacement method using a previously published TD1 structure after removing all the ligands and solvent molecules (PDB ID 4DRX) and the program PHASER in the PHENIX software package (Zwart *et al*, 2008). *Tt*TD1 crystallized in the space group P12_1_1 with two molecules of *Tt*TD1 in the asymmetric unit. The *Tt*TD1 structure was solved by molecular replacement using the T_βIII_D1 structure without the ligand as a model. Nucleotides were added to the model using eLBOW in PHENIX, and the structure was further refined through iterative rounds of model building in Coot (Emsley & Cowtan, 2004; Emsley *et al*, 2010) and PHENIX. The quality of the structure was assessed with MolProbity (Chen *et al*, 2010). Data collection and refinement statistics are presented in Appendix Table S2. Figures were prepared using PyMOL (The PyMOL Molecular Graphics System, version 1.4.1. Schrödinger).

### Cryo‐EM specimen preparation and data acquisition

A 4 μl of paclitaxel‐stabilized *Tt*‐MT at 10 μM was applied to a glow‐discharged C‐flat 2/2–4C grid (Protochips, Morrisville, NC) at room temperature and incubated on the grid for 1 min. The grid was subsequently blotted and plunge‐frozen using a Vitrobot Mark IV (Thermo Fisher Scientific) with the following setting: blot force of 5, blot time of 5 s, humidity of 100%, and temperature of 22°C. Micrographs of paclitaxel‐stabilized *Tt*‐MT were manually collected using a Tecnai Polara microscope (Thermo Fisher Scientific) operating at 300 kV, with a K2 summit direct electron detector (Gatan, CA, USA) and a quantum post‐column energy filter (Gatan) in zero‐loss imaging mode. A nominal defocus range of 1.0–3.5 μm and a final pixel size of 1.39 Å were used. The total dose was 45 e‐/Å^2^ over 50 frames, with the detector operating in counting mode at a rate of 5.8 e‐/pixel/s.

For the *Tt*‐MT samples used for 3D structure determination, a purified recombinant kinesin motor domain (*Plasmodium berghei* kinesin‐8B motor domain; PBANKA_0202700, residues 760–1,130) was bound to the MTs to act as a fiducial to facilitate image processing. Thus, after incubation of 10 μM paclitaxel‐stabilized *Tt*‐MT MTs on a C‐flat grid for 1 min, 3.5 μl of MTs were removed by pipetting to leave a thin film, followed by application of 3.5 μl of 50 uM purified kinesin‐8B motor domain, followed by 30 s incubation at room temperature. 3.5 μl of sample was removed from the grid by pipetting, followed by a second application of 3.5 μl of 50 μM kinesin, a further 30 s incubation, and the grid was then vitrified using the conditions above. The kinesin was prepared as follows. The *P. berghei* kinesin‐8B motor domain in the pNIC28Bsa4 vector encoding an N‐terminal His_6_ tag was transformed into *E. coli* BL21(DE3)* competent cells (Invitrogen), bacterial cultures were grown in large scale to OD 0.6–0.8 at 37°C and recombinant protein expression was induced with 20 μM IPTG at 20°C for 12 h. Pelleted cells were resuspended in lysis buffer (20 mM Tris–HCl pH 7.5, 500 mM NaCl, 5 mM MgCl_2_) with EDTA‐free protease inhibitor (Roche), followed by sonication for 30 min and then centrifugation at 50,000 *g*, 4°C for 30 min. The His_6_‐tagged kinesin was purified using immobilized metal affinity chromatography (IMAC), followed by incubation with TEV protease for 12 h at 4°C to remove His_6_ tag. The protein was exchanged into low‐salt buffer (20 mM Tris–HCl pH7.5, 100 mM NaCl, 5 mM MgCl_2_) and subjected to a further reverse‐IMAC step, followed by ion exchange (HiTrap Q HP IEX column, GE Healthcare) to remove any residual bacterial proteins, which bind to the Q column. The Q column flow‐through was aliquoted and snap‐frozen for further use. Before cryo‐EM sample preparation, the purified motor domain was buffer exchanged into 80 mM PIPES pH 6.8, 1 mM MgCl_2_, 1 mM EGTA, and incubated with apyrase (Sigma) for 10 min at room temperature to remove nucleotides.

A total of 6,188 micrographs were collected using a Titan Krios (Thermo Fisher Scientific) with a K2 summit direct electron detector (Gatan, CA, USA) and a quantum post‐column energy filter (Gatan) operating in zero‐loss imaging mode. The microscope was operated at an accelerating voltage of 300 kV with nominal magnification of 130 K and pixel size of 1.05 Å. Using EPU (Thermo Fisher Scientific), 32 frames for each movie were collected with 8 s exposure time, 47.112 e^−^/Å^2^ electron exposure, 1.472 e^−^/ Å^2^ frame dose, and 6.794 e‐/pixel/s dose rate. The defocus range was from −0.5 to −2.5 μm.

### Image processing and 3D reconstruction

Frames 2 to 16 were motion‐corrected using MotionCor2 (Zheng *et al*, 2017). The parameters of the contrast transfer function (CTF) for each micrograph were determined using CTFFIND4 (Rohou & Grigorieff, 2015). About 76,240 particles with box size of 644*644 pixels and non‐overlapping region of 78 pixels were picked using EMAN2 (Tang *et al*, 2007). Protofilament (pf) number determination and global refinement were done using multi‐reference alignment in EMAN1 (Ludtke *et al*, 1999; Tang *et al*, 2007). All 14 pf particles were merged for further data processing. Seam search and local refinement with HP symmetry were performed in FREALIGN v9 (Lyumkis *et al*, 2013) as described previously (Alushin *et al*, 2014; Zhang & Nogales, 2015). The pseudo‐helical symmetry parameters of MTs were measured from the asymmetric (C1) reconstruction of the same dataset followed by “hsearch” script in IHRSR package (Egelman, 2010). No B factor was applied to the final reconstruction. The FSC was calculated for the whole map reconstructed from the odd and even half of the datasets using a cylindrical, soft‐edge Gaussian mask with an inner radius of 75 Å and outer radius of 145 Å (Appendix Fig S10A).

### Microtubule structure modeling and refinement

A porcine tubulin dimer model (from PDB ID 6EW0) was fitted into the electron density of *T. thermophila* microtubules using fitmap command in UCSF Chimera (Pettersen *et al*, 2004). The fitted model was subsequently changed to *T. thermophila* tubulin sequence using COOT. Model building and refinement were performed as described previously (Eshun‐Wilson *et al*, 2019). Briefly, we built a central ⍺β tubulin dimer in COOT and refined a 4X3 lattice of tubulins. Refinement was performed by iterative cycles of phenix.real_space_refine in PHENIX (Afonine *et al*, 2018) and manual model building in COOT (Appendix Table S1 and Fig S10B and C).

### Modeling and drug design

In order to model the colchicine‐bound conformation, *Tt*TD1 structure was superimposed to the colchicine‐bound bovine tubulin model (PDB ID 4O2B) in COOT. The amino acid residues and secondary structure elements of β‐tubulin were modeled manually using Coot to mimic the colchicine‐bound conformation of *Tt* Tubulin (Tt–TD1_c_). CA4 was manually docked in the *Tt*TD1_c_ using COOT and the ligand‐bound pocket was energy‐minimized using MAB forcefield in Moloc (Gerber Molecular Design, Switzerland, (Gerber & Muller, 1995)). The modeled CA4‐*Tt*TD1 structure was used for further drug optimization using COOT. We manually searched the Enamine REAL database (Enamine Ltd.) using CA4 as a search query and selected compounds having the desired linkers. Compounds were selected by modeled in the *Tt*TD1_c_ structure in COOT, followed by binding pocket minimization in Moloc. Compounds showing favorable binding poses (by visual inspection in Coot) were selected and purchased from Enamine.

### *Tetrahymena* growth inhibition assay

For the *T. thermophila* growth inhibition assay, fresh cultures were prepared by inoculating 250 ml of SPP growth medium containing Pen/Strep antibiotics with 200 μl of a stock culture, followed by incubation at 30°C and 100 rpm shaking. Actively growing cells at cell density of 100,000 cells/ml were distributed at 100 μl volume into a microtiter plate. A mosquito automatic pipetting robot (SPT Labtech) was used to add 0.1 μl volumes of drugs or DMSO control to each well, and samples were evaluated in triplicate. The microtiter plate lids were treated with 0.1% Triton X‐100 in 20% ethanol to prevent condensation. Covered plates were incubated at 30°C for 72 h; absorbance at 600 nm was read every 30 min by a PheraStar FSX plate reader (BMG LABTECH). Data were plotted using DataGraph software (Visual Data Tools).

### Microtubule growth reconstitution assay

Bright MT seeds were prepared from cytoskeleton porcine tubulin stocks. Samples contained 1 mg/ml final concentration each of unlabeled, X‐rhodamine‐labeled, and biotin‐labeled tubulin in BRB80 with 0.5 mM GMPCPP. This solution was incubated at 37°C for 30 min, and then, the seeds were pelleted by centrifugation at 16,000 *g* for 10 min. Pelleted seeds were resuspended in 40 µl of BRB80 and used on the day they were prepared.

Flow chambers for TIRF microscopy were prepared on glass slides with double‐sided tape and biotin‐PEG coverslips (MicroSurfaces Inc.). Chambers were first incubated with blocking solution (0.75% Pluronic F‐127, 5 mg/ml casein) for 5 min, then washed with BRB80‐casein (80 mM PIPES, 5 mM MgCl_2_, 1 mM EGTA, 1 mM DTT, 1 mg/ml casein). 0.5 mg/ml neutravidin was incubated for 2 min and then excess removed with washes in BRB80‐casein. 1:200 diluted GMPCPP seeds were then incubated for 2 min and the excess washed out again with BRB80‐casein. The final assay mix was prepared in BRB80‐casein with an oxygen‐scavenging system (20 mM glucose, 300 μg/ml glucose oxidase, 60 μg/ml catalase), 0.1% methylcellulose, 0.5 mM GTP, and tubulin to the desired final concentration. The tubulin mix consisted of Alexa488‐labeled and unlabeled tubulin at a ratio of 1:4 for *Tetrahymena* samples and 1:9 for porcine samples. A range of tubulin concentrations between 2.5 and 5 μM were assessed for *Tetrahymena* MT dynamics; 3 μM was identified as a suitable concentration for the porcine tubulin experiments.

To test the effect of parabulin and colchicine (Sigma), 1 μl of drug was added at the desired concentration to the final assay mix. As a control, 1 μl DMSO was added in “WT” experiments.

For data collection, an Eclipse Ti‐E inverted microscope was used with a CFI Apo TIRF 1.49 N.A. oil objective, Perfect Focus System, H‐TIRF module, LU‐N4 laser unit (Nikon) and a quad band filter set (Chroma). Temperature was maintained at 35°C using a microscope incubator (Okolab). Frames were collected every 2 s with 100 ms exposure for 10 min using a iXon DU888 Ultra EMCCD camera (Andor) through the NIS‐Elements AR Software (Nikon). Illumination was performed sequentially every 2 s at 561 nm for the X‐rhodamine‐labeled GMPCPP seeds and 488 nm to show the Alexa488‐labeled MTs that polymerized. Movies were then analyzed in Fiji with drift‐correction if necessary, using StackReg rigid body transformation. Kymographs extracted from the movies were used to calculate growth rate, length, and catastrophe frequency values. For each condition, data from two or more movies were analyzed.

### Tissue culture

Human foreskin fibroblast (HFF) cells were grown in D10+ media composed of DMEM (Dulbecco's modified Eagle medium) supplemented with 10% fetal bovine serum (FBS), 1% l‐glutamine, and 1% penicillin–streptomycin. RH strain (ATCC, #50174) *T. gondii* tachyzoites were serially passaged in confluent HFF cells in T25 flasks.

### Immunofluorescence assay

Human foreskin fibroblast cells were seeded onto 12‐mm circular glass coverslips in 6‐well dishes 2‐4 days prior to assays. These cells were exposed to DMSO (vehicle control), 40 µM combretastatin A4 or 40 µM parabulin for 20–120 min at 37°C. Samples were fixed with 4% paraformaldehyde for 10 min, permeabilized with 0.25% Triton‐X in PBS‐ for 10 min, and blocked for 30 min in 10% BSA in PBS. Coverslips were stained with a mouse monoclonal anti‐tubulin antibody (Sigma T9026, 1:2,000) for 1 h. After washing in PBS, coverslips were exposed to goat anti‐mouse Alexa Fluor 488 secondary antibody (Invitrogen A28175, 1:4,000) for 1 h. After washing in PBS, coverslips were briefly rinsed in distilled water and mounted onto glass slides using VectaShield with DAPI. Images were collected on a Zeiss Axiovert 200 M, and images were exported and adjusted in Adobe Photoshop CC 2019.

### Toxicity assay

Subconfluent conditions: Approximately 5 x 10^4^ HFF cells were plated into each well of a 24‐well dish and exposed to D10+ media alone or D10+ with DMSO, combretastatin A4 or parabulin for 3 days. The plates were processed with the *In Vitro* Toxicology Assay Kit, MTT based (Sigma‐Aldrich, TOX1), and A_570_ was measured in a SpectraMax I3X plate reader. Exported data were analyzed using Microsoft Excel. Confluent conditions: Approximately 5 × 10^4^ HFF cells were plated into each well of a 24‐well dish and grown to D10+ media alone. Once the cells were confluent, they were gently rinsed with PBS to remove the media and then D10+ with DMSO or parabulin was added to each well. The plates were processed with the *In Vitro* Toxicology Assay Kit, MTT based (Sigma‐Aldrich, TOX1), and A570 was measured in a SpectraMax I3X plate reader. Exported data were analyzed using Microsoft Excel.

### Invasion assay

Freshly lysed out RH‐GFP parasites (Kim *et al*, 2001) were filtered and used to assess HFF monolayer invasion under control and parabulin‐treated conditions using an in–out assay as described by(Sweeney *et al*, 2010). Samples were fixed with formyl saline without subsequent permeabilization and stained with an anti‐SAG1 monoclonal antibody (Thermo Fisher) detected with an anti‐mouse Alexa 488 secondary antibody. Extracellular, invading, and intracellular parasites were scored from 10 random microscope fields; three biological replicates with individual technical triplicates were quantified.

### Replication assay

Freshly lysed out RH‐GFP parasites (Kim *et al*, 2001) were filtered and diluted by ¼ in D10+ media. 2 ml of diluted parasite media was added to each coverslip and parasites were allowed to invade into the cells for 30 min at 37°C. After this, the cell monolayer was washed with PBS to remove uninvaded parasites. D10+ media alone or D10+ with DMSO, 40 µM combretastastin A4, or 40 µM parabulin was added to the dishes. Actively growing parasites in fibroblasts were imaged every 12 h over a 48‐h period using a Zeiss Axiovert 200 M and Axiovision camera to collect 10 random fields of view for each condition. Samples were stained with a 1:1,000 anti‐SAG1 mouse monoclonal antibody (Thermo Fisher) and detected with a 1:1,000 dilution of a goat anti‐mouse Alexa 594 secondary antibody (Thermo Fisher). Parasite replication states were manually quantified and tallied from 10 random microscope fields to compare the proportion of singlets, doublets, quadruplets, and 8+ rosettes. Data were displayed as the fraction of total vacuoles/field that contains singlet, doublet, quadruplet, or 8+ numbers. Three biological replicates with individual technical triplicates were quantified.

### Determination of EC_50_ values

HFF cells (ATCC, #SRC‐1041) were grown to confluency in 6‐well dishes and infected with 300 parasites/well. Individual wells were treated with varying concentrations of parabulin (5‐60 µM), and the plates were left undisturbed for 7 days at 37°C in a humidified 5% CO_2_ incubator. After one week, the plates were fixed (100% methanol, 5 min at room temperature) and a 5× crystal violet histological stain solution was added to each well. Plaques appear as irregular clear areas that interrupt the purple‐colored host cell monolayer. Grayscale photographic images of the plates were taken to allow for differentiation between cells (dark gray) and plaques (white). ImageJ’s “threshold” functionality was used to calculate plaque number, average size, and percent total area within that field. To generate the EC_50_ curve, normalized % total plaque area (relative to no drug controls) was plotted versus drug concentration using GraphPad Prism. The results represent the average of 18 readings (6 biological replicates, each with 3 technical replicates) ± standard error of the mean.

## Author contributions

NG and AS conceived the presented ideas, carried out experiments, and wrote the manuscript with the help of all authors who provided critical feedback. IA and NM conceived and performed the Toxoplasma parasite experiments. TL, FS, and AC collected and processed the electron microscopy data under CAM supervision. MB, FS, and AJR performed the TIRF experiments and analyzed the data. VE produced the Tetrahymena crystals and collected the X‐ray diffraction data.

## Conflict of interest

The authors declare that they have no conflict of interest.

## Supporting information



Appendix

Expanded View Figures PDF

## Data Availability

Structural data were deposited in the RCSB Protein Data Bank (www.rcsb.org) with the PDB IDs: 7PJE (https://doi.org/10.2210/pdb7PJE/pdb) and 7PJF (https://doi.org/10.2210/pdb7PJF/pdb). Patent: Gaillard N. and Sharma A., Paul Scherrer Institute, “Use of parabulin for targeting protozoan tubulin for the inhibition of protozoan replication”, EP20200188.

## References

[emmm202013818-bib-0001] Afonine PV , Poon BK , Read RJ , Sobolev OV , Terwilliger TC , Urzhumtsev A , Adams PD (2018) Real‐space refinement in PHENIX for cryo‐EM and crystallography. Acta Crystallogr D Struct Biol 74: 531–544 29872004 10.1107/S2059798318006551 PMC6096492

[emmm202013818-bib-0002] Akhmanova A , Steinmetz MO (2015) Control of microtubule organization and dynamics: two ends in the limelight. Nat Rev Mol Cell Biol 16: 711–726 26562752 10.1038/nrm4084

[emmm202013818-bib-0003] Alushin GM , Lander GC , Kellogg EH , Zhang R , Baker D , Nogales E (2014) High‐resolution microtubule structures reveal the structural transitions in alphabeta‐tubulin upon GTP hydrolysis. Cell 157: 1117–1129 24855948 10.1016/j.cell.2014.03.053 PMC4054694

[emmm202013818-bib-0004] Ayaz P , Munyoki S , Geyer EA , Piedra FA , Vu ES , Bromberg R , Otwinowski Z , Grishin NV , Brautigam CA , Rice LM (2014) A tethered delivery mechanism explains the catalytic action of a microtubule polymerase. Elife 3: e03069 25097237 10.7554/eLife.03069 PMC4145800

[emmm202013818-bib-0005] Bejon PA , Bannister LH , Fowler RE , Fookes RE , Webb SE , Wright A , Mitchell GH (1997) A role for microtubules in Plasmodium falciparum merozoite invasion. Parasitology 114(Pt 1): 1–6 9289695 10.1017/s0031182096008050

[emmm202013818-bib-0006] Chaaban S , Jariwala S , Hsu CT , Redemann S , Kollman JM , Muller‐Reichert T , Sept D , Bui KH , Brouhard GJ (2018) The structure and dynamics of *C. elegans* tubulin reveals the mechanistic basis of microtubule growth. Dev Cell 47: 191–204.e198 30245157 10.1016/j.devcel.2018.08.023

[emmm202013818-bib-0007] Chen VB , Arendall 3rd WB , Headd JJ , Keedy DA , Immormino RM , Kapral GJ , Murray LW , Richardson JS , Richardson DC (2010) MolProbity: all‐atom structure validation for macromolecular crystallography. Acta Crystallogr D Biol Crystallogr 66: 12–21 20057044 10.1107/S0907444909042073 PMC2803126

[emmm202013818-bib-0008] Dorrell RG , Butterfield ER , Nisbet RE , Howe CJ (2013) Evolution: unveiling early alveolates. Curr Biol 23: R1093–R1096 24355784 10.1016/j.cub.2013.10.055

[emmm202013818-bib-0009] Dumontet C , Jordan MA (2010) Microtubule‐binding agents: a dynamic field of cancer therapeutics. Nat Rev Drug Discov 9: 790–803 20885410 10.1038/nrd3253 PMC3194401

[emmm202013818-bib-0010] Egelman EH (2010) Reconstruction of helical filaments and tubes. Methods Enzymol 482: 167–183 20888961 10.1016/S0076-6879(10)82006-3 PMC3245864

[emmm202013818-bib-0011] Emsley P , Cowtan K (2004) Coot: model‐building tools for molecular graphics. Acta Crystallogr D Biol Crystallogr 60: 2126–2132 15572765 10.1107/S0907444904019158

[emmm202013818-bib-0012] Emsley P , Lohkamp B , Scott WG , Cowtan K (2010) Features and development of Coot. Acta Crystallogr D Biol Crystallogr 66: 486–501 20383002 10.1107/S0907444910007493 PMC2852313

[emmm202013818-bib-0013] Eshun‐Wilson L , Zhang R , Portran D , Nachury MV , Toso DB , Lohr T , Vendruscolo M , Bonomi M , Fraser JS , Nogales E (2019) Effects of alpha‐tubulin acetylation on microtubule structure and stability. Proc Natl Acad Sci U S A 116: 10366–10371 31072936 10.1073/pnas.1900441116 PMC6535015

[emmm202013818-bib-0014] Fennell B , Naughton J , Barlow J , Brennan G , Fairweather I , Hoey E , McFerran N , Trudgett A , Bell A (2008) Microtubules as antiparasitic drug targets. Expert Opin Drug Discov 3: 501–518 23484923 10.1517/17460441.3.5.501

[emmm202013818-bib-0015] Fernández E , Castellote MI , Chaparro MJ , Díaz B , Fernández J , Gordo M , de las Heras L , León ML , Rueda L , Calderón F (2019) Chapter One ‐ A decade of malaria phenotypic screenings: Key lessons on the discovery and development of new antimalarial drugs. In: Annual reports in medicinal chemistry, Chibale K (ed.), pp 1–23. Cambridge, MA: Academic Press

[emmm202013818-bib-0016] Gaspari R , Prota AE , Bargsten K , Cavalli A , Steinmetz MO (2017) Structural basis of cis‐ and trans‐combretastatin binding to tubulin. Chem 2: 102–113

[emmm202013818-bib-0017] Gerber PR , Muller K (1995) MAB, a generally applicable molecular force field for structure modelling in medicinal chemistry. J Comput Aided Mol Des 9: 251–268 7561977 10.1007/BF00124456

[emmm202013818-bib-0018] Grisham R , Ky B , Tewari KS , Chaplin DJ , Walker J (2018) Clinical trial experience with CA4P anticancer therapy: focus on efficacy, cardiovascular adverse events, and hypertension management. Gynecol Oncol Res Pract 5: 1–10 29318022 10.1186/s40661-017-0058-5 PMC5756341

[emmm202013818-bib-0019] Haldar K , Bhattacharjee S , Safeukui I (2018) Drug resistance in Plasmodium. Nat Rev Microbiol 16: 156–170 29355852 10.1038/nrmicro.2017.161 PMC6371404

[emmm202013818-bib-0020] Huynh MH , Rabenau KE , Harper JM , Beatty WL , Sibley LD , Carruthers VB (2003) Rapid invasion of host cells by Toxoplasma requires secretion of the MIC2‐M2AP adhesive protein complex. EMBO J 22: 2082–2090 12727875 10.1093/emboj/cdg217 PMC156089

[emmm202013818-bib-0021] Kabsch W (2010a) Integration, scaling, space‐group assignment and post‐refinement. Acta Crystallogr D Biol Crystallogr 66: 133–144 20124693 10.1107/S0907444909047374 PMC2815666

[emmm202013818-bib-0022] Kabsch W (2010b) Xds. Acta Crystallogr D Biol Crystallogr 66: 125–132 20124692 10.1107/S0907444909047337 PMC2815665

[emmm202013818-bib-0023] Kim K , Eaton MS , Schubert W , Wu S , Tang J (2001) Optimized expression of green fluorescent protein in Toxoplasma gondii using thermostable green fluorescent protein mutants. Mol Biochem Parasitol 113: 309–313 11295185 10.1016/s0166-6851(01)00212-2

[emmm202013818-bib-0024] King RL , Beams HW (1940) A comparison of the effects of colchicine on division in protozoa and certain other cells. J Cell Comp Physiol 15: 252–254

[emmm202013818-bib-0025] Knossow M , Campanacci V , Khodja LA , Gigant B (2020) The mechanism of tubulin assembly into microtubules: insights from structural. Studies 23: 101511 10.1016/j.isci.2020.101511 PMC7491153 32920486

[emmm202013818-bib-0026] La Sala G , Olieric N , Sharma A , Viti F , de Asis Balaguer Perez F , Huang L , Tonra JR , Lloyd GK , Decherchi S , Díaz JF *et al* (2019) Structure, thermodynamics, and kinetics of plinabulin binding to two tubulin isotypes. Chem 5: 2969–2986

[emmm202013818-bib-0027] Ludtke SJ , Baldwin PR , Chiu W (1999) EMAN: semiautomated software for high‐resolution single‐particle reconstructions. J Struct Biol 128: 82–97 10600563 10.1006/jsbi.1999.4174

[emmm202013818-bib-0028] Lyons‐Abbott S , Sackett DL , Wloga D , Gaertig J , Morgan RE , Werbovetz KA , Morrissette NS (2010) alpha‐Tubulin mutations alter oryzalin affinity and microtubule assembly properties to confer dinitroaniline resistance. Eukaryot Cell 9: 1825–1834 20870876 10.1128/EC.00140-10 PMC3008275

[emmm202013818-bib-0029] Lyumkis D , Brilot AF , Theobald DL , Grigorieff N (2013) Likelihood‐based classification of cryo‐EM images using FREALIGN. J Struct Biol 183: 377–388 23872434 10.1016/j.jsb.2013.07.005 PMC3824613

[emmm202013818-bib-0030] Ma C , Li C , Ganesan L , Oak J , Tsai S , Sept D , Morrissette NS (2007) Mutations in alpha‐tubulin confer dinitroaniline resistance at a cost to microtubule function. Mol Biol Cell 18: 4711–4720 17881728 10.1091/mbc.E07-04-0379 PMC2096588

[emmm202013818-bib-0031] Ma C , Tran J , Li C , Ganesan L , Wood D , Morrissette N (2008) Secondary mutations correct fitness defects in Toxoplasma gondii with dinitroaniline resistance mutations. Genetics 180: 845–856 18780736 10.1534/genetics.108.092494 PMC2567385

[emmm202013818-bib-0032] Massarotti A , Coluccia A , Silvestri R , Sorba G , Brancale A (2012) The tubulin colchicine domain: a molecular modeling perspective. ChemMedChem 7: 33–42 21990124 10.1002/cmdc.201100361

[emmm202013818-bib-0033] Menard D , Dondorp A (2017) Antimalarial drug resistance: a threat to malaria elimination. Cold Spring Harb Perspect Med 7: a025619 28289248 10.1101/cshperspect.a025619 PMC5495053

[emmm202013818-bib-0034] Mooney CJ , Nagaiah G , Fu P , Wasman JK , Cooney MM , Savvides PS , Bokar JA , Dowlati A , Wang D , Agarwala SS *et al* (2009) A phase II trial of fosbretabulin in advanced anaplastic thyroid carcinoma and correlation of baseline serum‐soluble intracellular adhesion molecule‐1 with outcome. Thyroid 19: 233–240 19265494 10.1089/thy.2008.0321 PMC2913806

[emmm202013818-bib-0035] Morrissette N (2015) Targeting Toxoplasma tubules: tubulin, microtubules, and associated proteins in a human pathogen. Eukaryot Cell 14: 2–12 25380753 10.1128/EC.00225-14 PMC4279016

[emmm202013818-bib-0036] Morrissette NS , Sibley LD (2002) Disruption of microtubules uncouples budding and nuclear division in Toxoplasma gondii. J Cell Sci 115: 1017–1025 11870220 10.1242/jcs.115.5.1017

[emmm202013818-bib-0037] Pecqueur L , Duellberg C , Dreier B , Jiang Q , Wang C , Pluckthun A , Surrey T , Gigant B , Knossow M (2012) A designed ankyrin repeat protein selected to bind to tubulin caps the microtubule plus end. Proc Natl Acad Sci U S A 109: 12011–12016 22778434 10.1073/pnas.1204129109 PMC3409770

[emmm202013818-bib-0038] Pettersen EF , Goddard TD , Huang CC , Couch GS , Greenblatt DM , Meng EC , Ferrin TE (2004) UCSF Chimera–a visualization system for exploratory research and analysis. J Comput Chem 25: 1605–1612 15264254 10.1002/jcc.20084

[emmm202013818-bib-0039] Prota AE , Danel F , Bachmann F , Bargsten K , Buey RM , Pohlmann J , Reinelt S , Lane H , Steinmetz MO (2014) The novel microtubule‐destabilizing drug BAL27862 binds to the colchicine site of tubulin with distinct effects on microtubule organization. J Mol Biol 426: 1848–1860 24530796 10.1016/j.jmb.2014.02.005

[emmm202013818-bib-0040] Ravelli RB , Gigant B , Curmi PA , Jourdain I , Lachkar S , Sobel A , Knossow M (2004) Insight into tubulin regulation from a complex with colchicine and a stathmin‐like domain. Nature 428: 198–202 15014504 10.1038/nature02393

[emmm202013818-bib-0041] Rohou A , Grigorieff N (2015) CTFFIND4: fast and accurate defocus estimation from electron micrographs. J Struct Biol 192: 216–221 26278980 10.1016/j.jsb.2015.08.008 PMC6760662

[emmm202013818-bib-0042] Sharma A , Aher A , Dynes N , Frey D , Katrukha E , Jaussi R , Grigoriev I , Croisier M , Kammerer R , Akhmanova A *et al* (2016) Centriolar CPAP/SAS‐4 imparts slow processive microtubule growth. Dev Cell 37: 362–376 27219064 10.1016/j.devcel.2016.04.024 PMC4884677

[emmm202013818-bib-0043] Sharma A , Saez‐Calvo G , Olieric N , de Asis BF , Barasoain I , Lamberth C , Diaz JF , Steinmetz MO (2017) Quinolin‐6‐yloxyacetamides are microtubule destabilizing agents that bind to the colchicine site of tubulin. Int J Mol Sci 18: 1336 10.3390/ijms18071336 PMC5535829 28640209

[emmm202013818-bib-0044] Steinmetz MO , Prota AE (2018) Microtubule‐targeting agents: strategies to hijack the cytoskeleton. Trends Cell Biol 28: 776–792 29871823 10.1016/j.tcb.2018.05.001

[emmm202013818-bib-0045] Sweeney KR , Morrissette NS , LaChapelle S , Blader IJ (2010) Host cell invasion by Toxoplasma gondii is temporally regulated by the host microtubule cytoskeleton. Eukaryot Cell 9: 1680–1689 20435700 10.1128/EC.00079-10 PMC2976295

[emmm202013818-bib-0046] Tang G , Peng L , Baldwin PR , Mann DS , Jiang W , Rees I , Ludtke SJ (2007) EMAN2: an extensible image processing suite for electron microscopy. J Struct Biol 157: 38–46 16859925 10.1016/j.jsb.2006.05.009

[emmm202013818-bib-0047] Telley IA , Bieling P , Surrey T (2011) Reconstitution and quantification of dynamic microtubule end tracking *in vitro* using TIRF microscopy. Methods Mol Biol 777: 127–145 21773926 10.1007/978-1-61779-252-6_10

[emmm202013818-bib-0048] Ti SC , Pamula MC , Howes SC , Duellberg C , Cade NI , Kleiner RE , Forth S , Surrey T , Nogales E , Kapoor TM (2016) Mutations in human tubulin proximal to the kinesin‐binding site alter dynamic instability at microtubule plus‐ and minus‐ends. Dev Cell 37: 72–84 27046833 10.1016/j.devcel.2016.03.003 PMC4832424

[emmm202013818-bib-0049] Ullah I , Gahalawat S , Booshehri LM , Niederstrasser H , Majumdar S , Leija C , Bradford JM , Hu B , Ready JM , Wetzel DM (2020) An antiparasitic compound from the medicines for malaria venture pathogen box promotes leishmania tubulin polymerization. ACS Infect Dis 6: 2057–2072 32686409 10.1021/acsinfecdis.0c00122 PMC8059355

[emmm202013818-bib-0050] Uwimana A , Legrand E , Stokes BH , Ndikumana J‐L , Warsame M , Umulisa N , Ngamije D , Munyaneza T , Mazarati J‐B , Munguti K *et al* (2020) Emergence and clonal expansion of *in vitro* artemisinin‐resistant Plasmodium falciparum kelch13 R561H mutant parasites in Rwanda. Nat Med 26: 1602–1608 32747827 10.1038/s41591-020-1005-2 PMC7541349

[emmm202013818-bib-0051] Vemu A , Atherton J , Spector JO , Szyk A , Moores CA , Roll‐Mecak A (2016) Structure and dynamics of single‐isoform recombinant neuronal human tubulin. J Biol Chem 291: 12907–12915 27129203 10.1074/jbc.C116.731133 PMC4933209

[emmm202013818-bib-0052] Wang Y , Zhang H , Gigant B , Yu Y , Wu Y , Chen X , Lai Q , Yang Z , Chen Q , Yang J (2016) Structures of a diverse set of colchicine binding site inhibitors in complex with tubulin provide a rationale for drug discovery. FEBS J 283: 102–111 26462166 10.1111/febs.13555

[emmm202013818-bib-0053] Zhang R , Nogales E (2015) A new protocol to accurately determine microtubule lattice seam location. J Struct Biol 192: 245–254 26424086 10.1016/j.jsb.2015.09.015 PMC4634897

[emmm202013818-bib-0054] Zheng SQ , Palovcak E , Armache JP , Verba KA , Cheng Y , Agard DA (2017) MotionCor2: anisotropic correction of beam‐induced motion for improved cryo‐electron microscopy. Nat Methods 14: 331–332 28250466 10.1038/nmeth.4193 PMC5494038

[emmm202013818-bib-0055] Zwart PH , Afonine PV , Grosse‐Kunstleve RW , Hung LW , Ioerger TR , McCoy AJ , McKee E , Moriarty NW , Read RJ , Sacchettini JC *et al* (2008) Automated structure solution with the PHENIX suite. Methods Mol Biol 426: 419–435 18542881 10.1007/978-1-60327-058-8_28

