# Supplementary material for: Inhibiting parasite proliferation using a rationally designed anti‐tubulin agent
Source: EMBO Mol Med. 2021 Oct 18;13(11):e13818. doi: 10.15252/emmm.202013818 (PMC8573600; doi:10.15252/emmm.202013818)
Supplement: Supplementary file 1 — Appendix [file EMMM-13-e13818-s001.pdf]

Table of content

Appendix Figure S1
T. thermophila MT growth imaged by TIRF microscopy.

Appendix Figure S2
Cryo-electron microscopy of *T. thermophila* MTs.

Appendix Figure S3
Multiple sequence alignment of α-tubulin.

Appendix Figure S4
Multiple sequence alignment of β-tubulin.

Appendix Figure S5
Testing of ligands binding at different zones of the colchicine-site using *T. thermophila* growth inhibition assay.

Appendix Figure S6
Parabulin toxicity and effects on *T. gondii* invasion.

Appendix Table S1
X-ray data collection and refinement statistics.

Appendix Table S2
Electron microscopy data collection and refinement statistics.

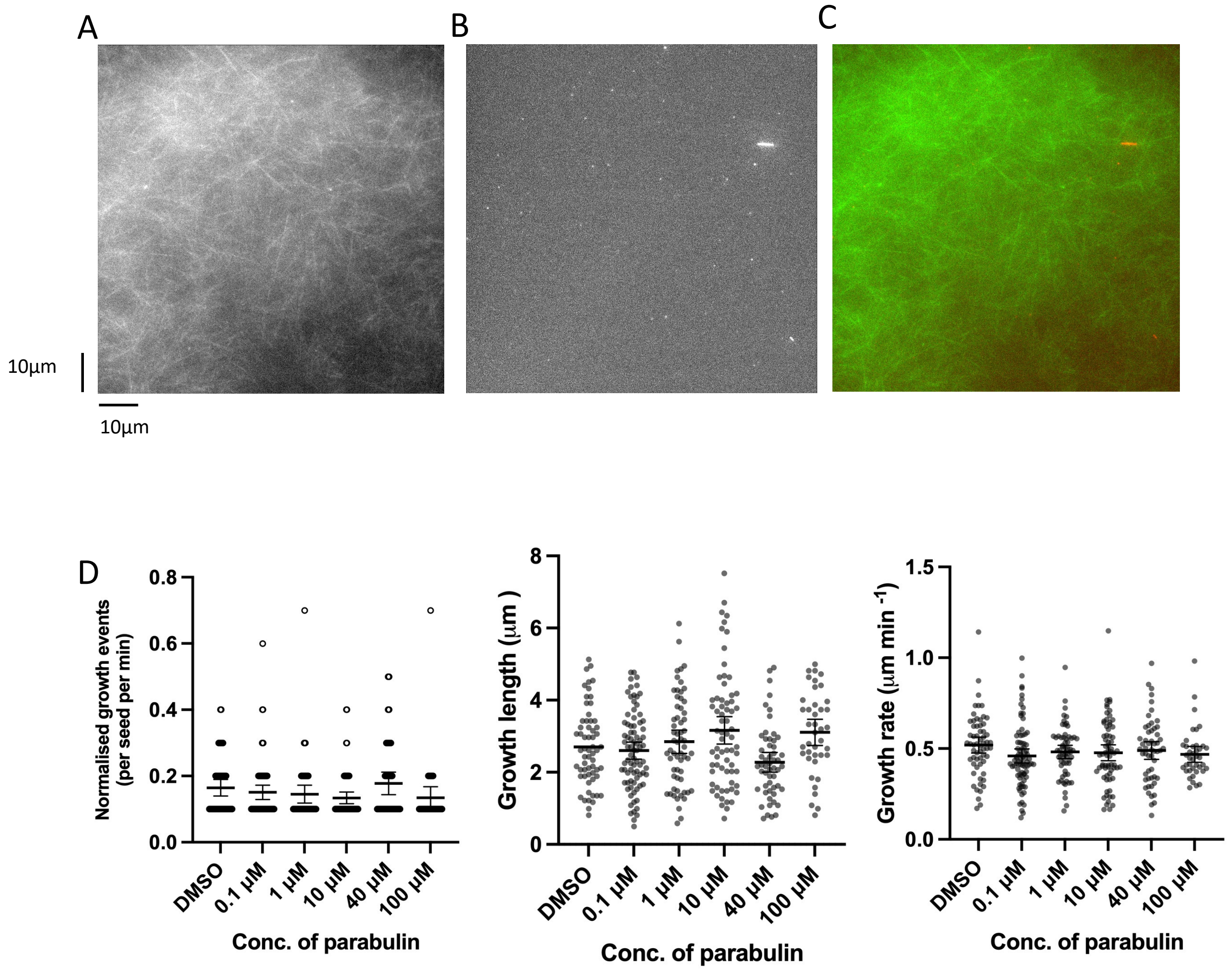

Appendix Figure S1: ***T. thermophila*** **MT growth imaged by TIRF microscopy**.

(A) 488nm fluorescence from labelled Tt-tubulin. (B) 561nm fluorescence from X-rhodamine labelled GMPCPP-MT porcine tubulin seeds. (C) Composite image of two series superimposed. *Tt*-MT nucleation was spontaneous (i.e. not all growth emanated from seeds) and microtubules filled the field of view. (D) Scatter plots showing the distribution of porcine brain MT (Cytoskeleton, Catalog no.T240) normalised growth events (left), growth rates (centre) and growth lengths (right) in TIRF microscopy based MT dynamics reconstitution assays in the presence of increasing concentrations of parabulin. DMSO represents the control and concentrations of 0.1uM,1uM,10uM, 40uM and 100uM of parabulin were used in the experiments. Error bars represent the mean +/- 95% CI. For each concentration, data is obtained from at least 2 independent experiments. For growth rates and growth lengths, n=61(DMSO), n= 81(0.1uM parabulin), n= 61(1uM parabulin), n=68 (10uM parabulin), n=54 (40uM parabulin) and n=40 (100uM parabulin) and for growth events n=47 (DMSO), n=69 (0.1uM parabulin), n=53 (1uM parabulin), n=62 (10uM parabulin), n=45 (40uM parabulin) and n= 38 (100uM parabulin).

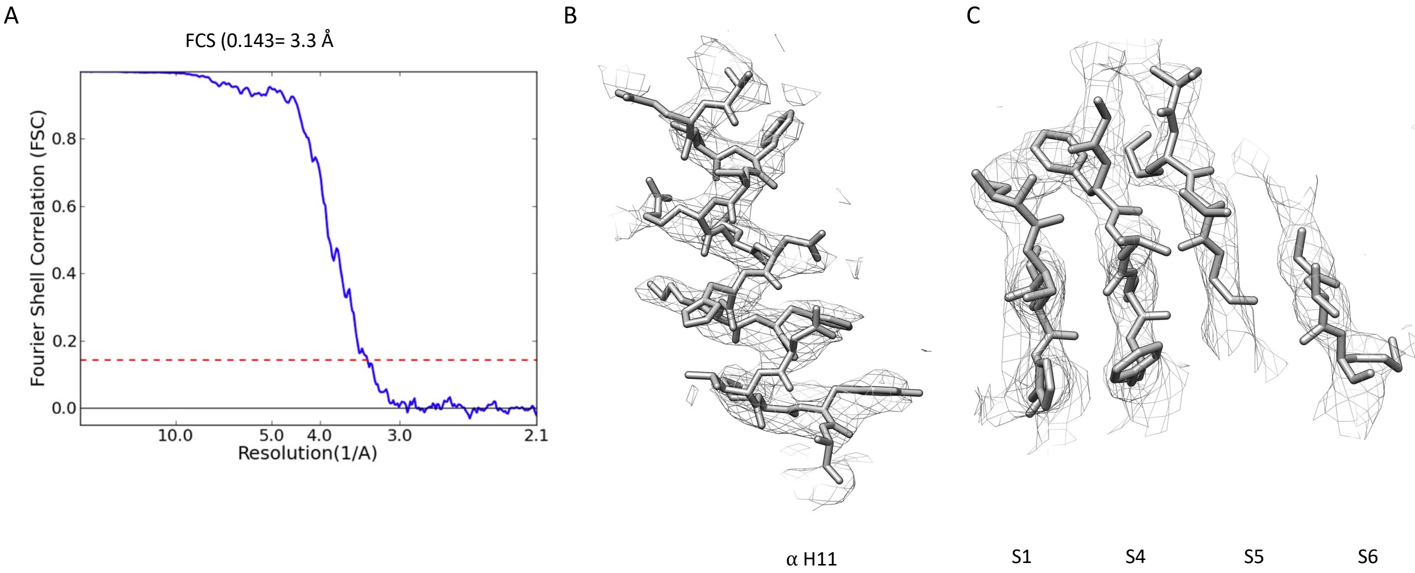

Appendix Figure S2: Cryo-electron microscopy of *T. thermophila* MTs.

(A) Fourier Shell Correlation (FSC) curve of the reconstruction of paclitaxel stabilized *T. thermophila* MTs. The FSC curve of the reconstruction shows that the overall resolution is 3.3 Å (FSC 0.143). (B) Density fitting showing quality of resolved α-helix H11 and (C) β-strand separation (S1, S4-S6) of α-tubulin.

α-tubulin

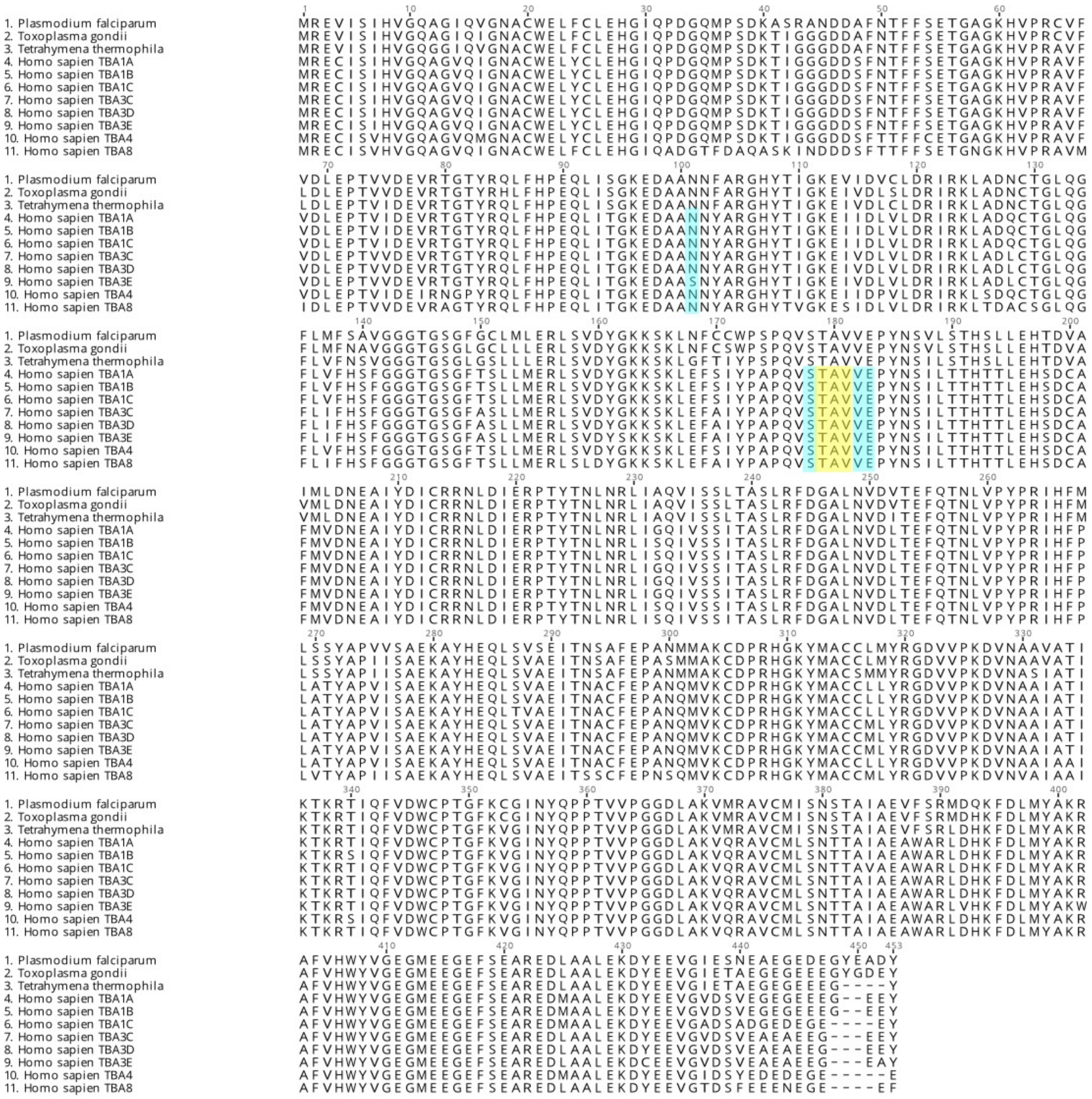

Appendix Figure S3: Multiple sequence alignment of α-tubulin.

UniProtKB/Swiss-Prot sequence accession identifiers are as follows: Plasmodium falciparum, TBA_PLAFK; Toxoplasma gondii, TBA_TOXGO; Tetrahymena thermophila, TBA_TETTH; Homo sapiens, TBA1A, TBA1B, TBA1C, TBA3C, TBA3D, TBA3E, TBA4, and TBA8. Residues within 6 Å of combretastatin A4 are highlighted in yellow, and additional residues within 6 Å of colchicine are highlighted in aqua.

β-tubulin

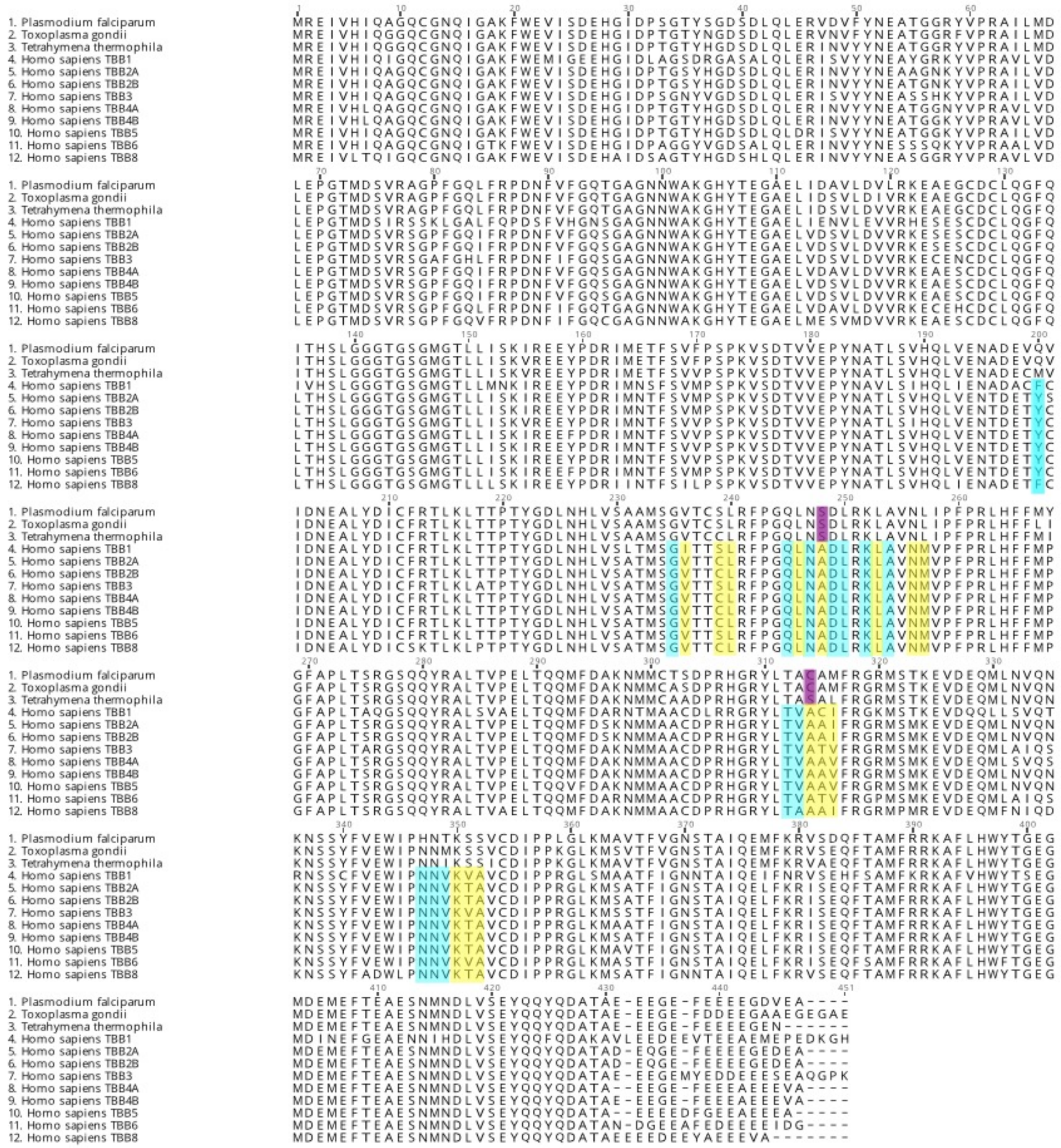

Appendix Figure S4: Multiple sequence alignment of b-tubulin.

UniProtKB/Swiss-Prot sequence accession identifiers are as follows: Plasmodium falciparum, TBB_PLAFK; Toxoplasma gondii, TBB_TOXGO; Tetrahymena thermophila, TBB_TETTH; Homo sapiens, TBB1, TBB2A, TBB2B, TBB3, TBB4A, TBB4B, TBB5, TBB6, and TBB8. Residues within 6 Å of combretastatin A4 are highlighted in yellow, and additional residues within 6 Å of colchicine are highlighted in aqua.

A

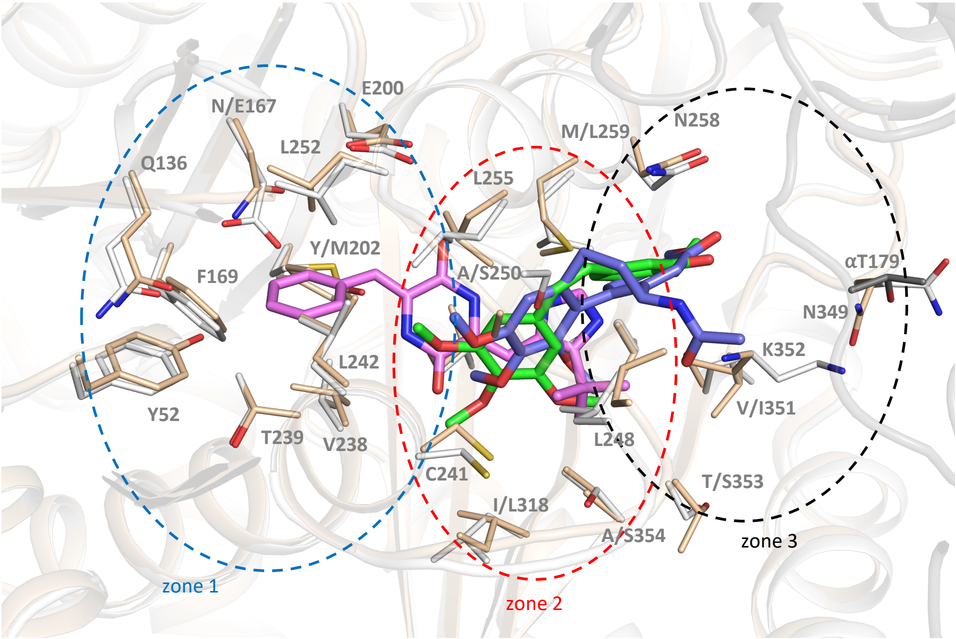

B

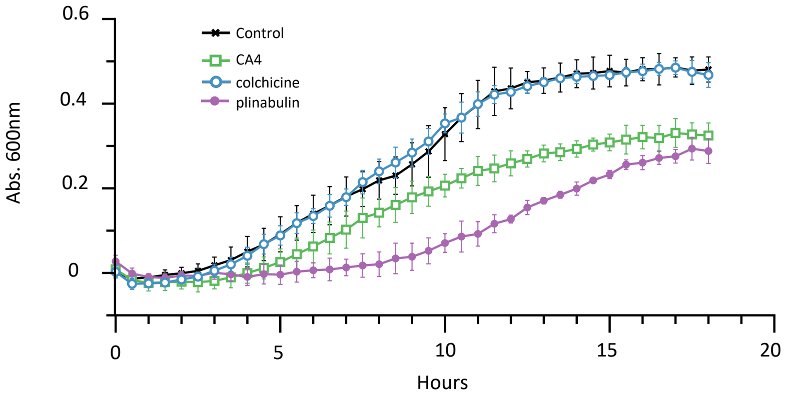

Appendix Figure S5: **Testing of ligands binding at different zones of the colchicine-site using *T. thermophila* growth inhibition assay.**

(A) Structural superposition showing representative drugs binding at the distinct zones of colchicine site of *Tt*-tubulin. The three distinct zones of the colchicine-site are highlighted by colored dotted circles. Plinabulin (magenta), colchicine (blue) and CA4 (green). (B) Plot showing the time course of *T. thermophila* cell growth in the presence of the indicated drugs at 20µM concentration (error bars corresponding to the standard deviation of triplicate measurements). Control experiments are done with DMSO alone.

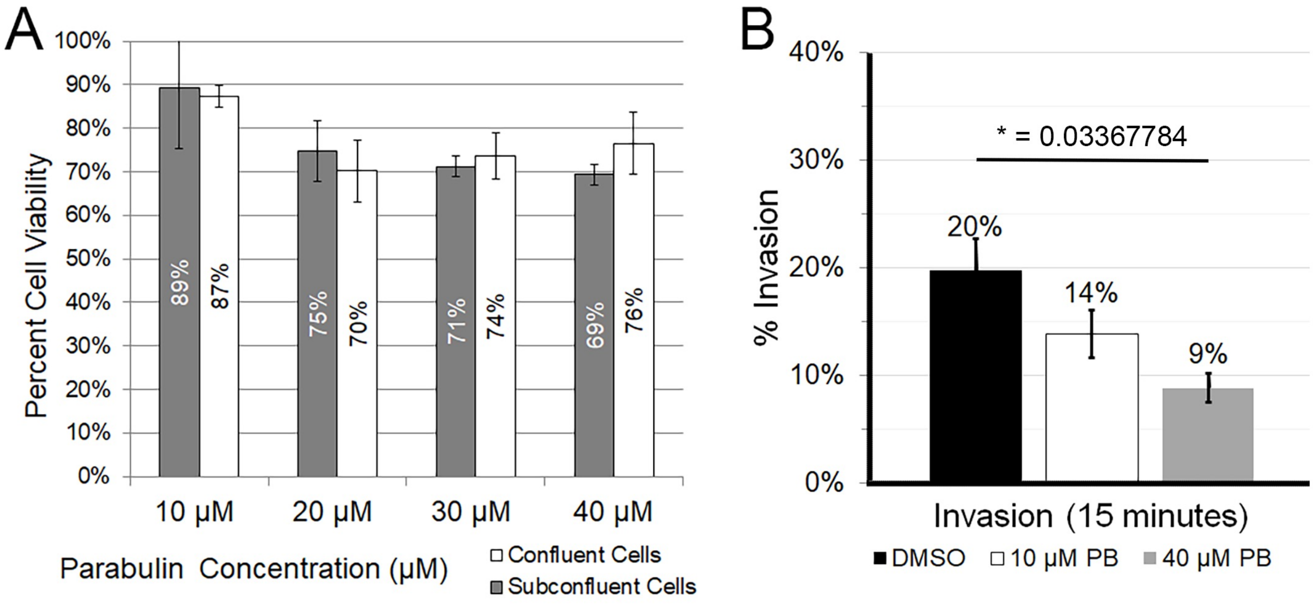

Appendix Figure S6: Parabulin toxicity and effects on T. gondii invasion.

(A) MTT assays for sub-confluent (replicating) and confluent (contact inhibited) human fibroblasts at a range of parabulin concentrations, with values normalized to untreated control cultures. The sub-confluent cell data represent the average of 12 readings (4 biological replicates with 3 technical replicates of each treatment) ± standard error of the mean. The confluent cell data represent the average of 9 readings (3 biological replicates with 3 technical replicates of each treatment) ± standard error of the mean. (B) Parabulin treatment reduces tachyzoite invasion of host cells relative to untreated controls. The fraction of intracellular parasites to total attached parasites observed in 10 fields of view is represented for three biological replicates, fixed 15 min after parasite addition to host monolayers. Error bars represent standard error of the mean. Statistical analysis was performed using Microsoft Excel. Student's t-test was used to determine statistical significance among the different conditions tested. A P value of less than 0.05 was considered significant.

Grid type	C-Flat 2/2-4C
Microscope	Krios
Detector and mode	K2 counting mode
Collection software	EPU
Magnification	130K
Voltage(Kv)	300
Electron exposure (e^-/ Å^2)	47
Exposure time(s)	8
Dose rate (e-/pixel/s)	6.8
Frame number	32
Frame dose (e^-/ Å^2)	1.472
Defocus range(um)	-.5 to -2.5
Pixel size(Å)	1.05
Micrograph number	6188
Computing software	MotionCor2, EMAN, FREALIGN
Particle number for final reconstruction	27,858
Helical symmetry (N number)	14
Helical parameter (Å, dimer rise)	8.76
Helical parameter (° , dimer turn)	-25.77
Map resolution (Å, FSC 0.143)	3.3
RMS bonds (Å)	0.007
RMS angles (°)	1.163
MolProbility score	2.27
Clashscore	7.90
Ramachandran plot (%)	
Favored	92.46
Allowed	7.54
Disallowed	0.00

Appendix Table S1: X-ray data collection and refinement statistics.

Table S2. X-Ray Data Collection and Refinement Statistics

Data Collection[a]	Tt-TD1	Hs-$T_{\beta III}$D1
Wavelength, Å	1	1
Space group	P 1 21 1	P 1 21 1
Resolution range, Å	45.98 - 1.75 (1.813 - 1.75)	45.66 - 1.862 (1.929 - 1.862)
Unit cell a, b, c (Å) α, β, γ (°)	52.13 183.92 118.323 90 92.44 90	73.784 91.312 82.657 90 97.546 90
No. of total reflections	1304001 (121833)	621604 (60067)
No. of unique reflections	219446 (20372)	90757 (8956)
Completeness (%)	98.37 (91.63)	99.80 (98.65)
Multiplicity	5.9 (6.0)	6.8 (6.7)
Mean I/sigma(I)	12.84 (1.57)	19.16 (1.75)
R-merge	0.07281 (1.177)	0.0701 (1.22)
R-meas	0.08004 (1.287)	0.07585 (1.322)
R-pim	0.03267 (0.5162)	0.02872 (0.5035)
CC1/2[b]	0.997 (0.796)	0.999 (0.782)
CC*	0.999 (0.942)	1 (0.937)
Refinement		
R-work	0.1779 (0.3342)	0.1837 (0.3335)
R-free	0.2132 (0.3585)	0.2185 (0.3774)
Macromolecules	15192	7766
Ligands	131	66
Protein residues	1946	1003
RMS (bonds) (Å)	0.010	0.016
RMS (angles) (°)	1.10	1.51
Ramachandran favored (%)[c]	97.76	97.37
Ramachandran outliers (%)[c]	0.10	0.00
B-factors		
Average B-factor	46.00	40.01
Macromolecules	45.99	39.89
Ligands	35.75	27.33
Solvent	47.37	43.97
Number of TLS groups	37	24

[a] Highest resolution shell statistics are in parentheses.
[b] As defined by Karplus and Diederichs (Karplus and Diederichs, 2012)
[c] As defined by MolProbity (Chen et al., 2010)

Appendix Table S2: Electron microscopy data collection and refinement statistics.
