# Supplementary material for: Inhibiting parasite proliferation using a rationally designed anti‐tubulin agent
Source: EMBO Mol Med. 2021 Oct 18;13(11):e13818. doi: 10.15252/emmm.202013818 (PMC8573600; doi:10.15252/emmm.202013818)
Supplement: Supplementary file 2 — Expanded View Figures PDF [file EMMM-13-e13818-s003.pdf]

Expanded View Figures

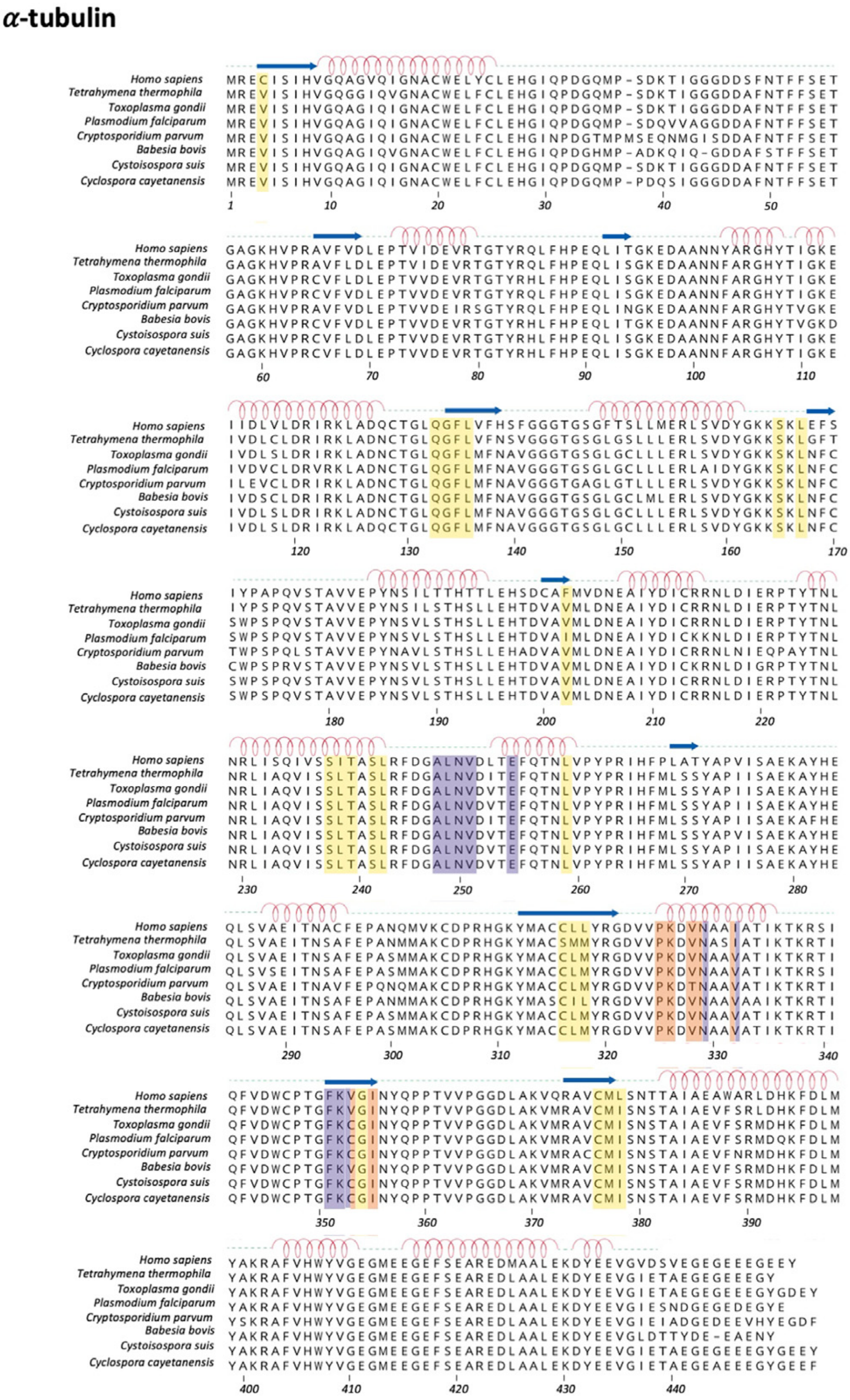

Figure EV1.

◀ **Figure EV1. Multiple sequence alignment of α-tubulin and mapping of known drug-binding sites.**

UniProtKB/Swiss-Prot sequence accession identifiers are as follows: *Homo sapiens*, Hs_TBA1B; *Tetrahymena thermophila*, Tetrahymena_α-Tubulin (P41351); *Toxoplasma gondii*, TGME49_316400; *Plasmodium falciparum*, PF3D7_0422300; *Cryptosporidium parvum*, cgd4_2860; *Babesia bovis*, BBOV_III002820; *Cystoisospora suis* CSUI_008696; *Cyclospora cayetanensis*, cyc_07446. Residues defining the following drug-binding sites are highlighted: yellow, pironetin site; orange, vinblastine site; violet, eribulin site.

 © 2021 The Authors

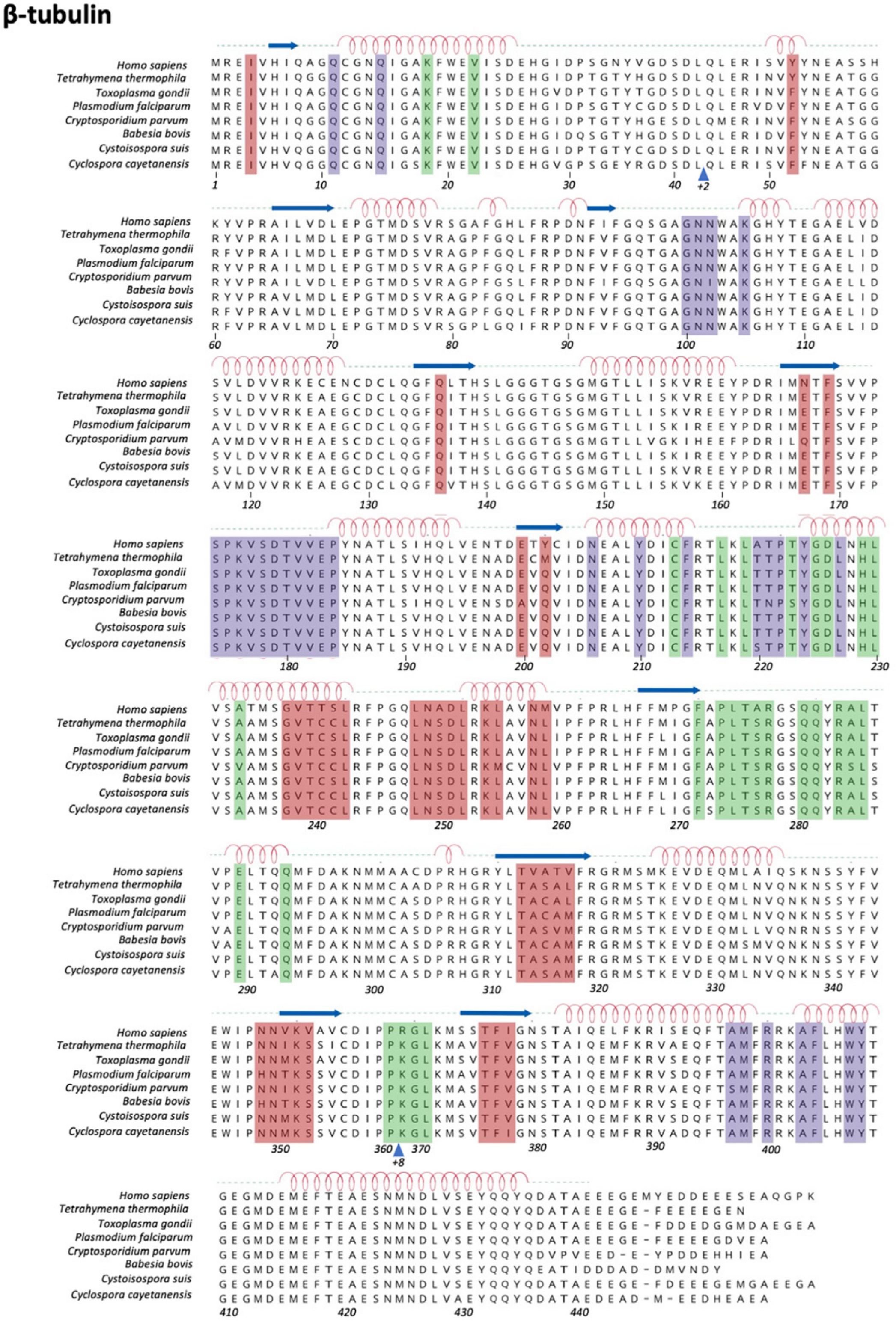

Figure EV2. Multiple sequence alignment of β-tubulin and mapping of known drug-binding sites.

UniProtKB/Swiss-Prot sequence accession identifiers are as follows: *Homo sapiens*, Hs_TBB3; *Tetrahymena thermophila*, Tetrahymena_BTUB1 (P41352); *Toxoplasma gondii*, TGME49_221620; *Plasmodium falciparum*, PF3D7_1008700; *Cryptosporidium parvum*, cgd6_4760; *Babesia bovis*, BBOV_III004850; *Cystoisospora suis*, CSUI_006169; *Cyclospora cayetanensis*, cyc_00127. Residues defining the following drug-binding sites are highlighted: red, colchicine site; green, taxane site; violet, maytansine site.

© 2021 The Authors

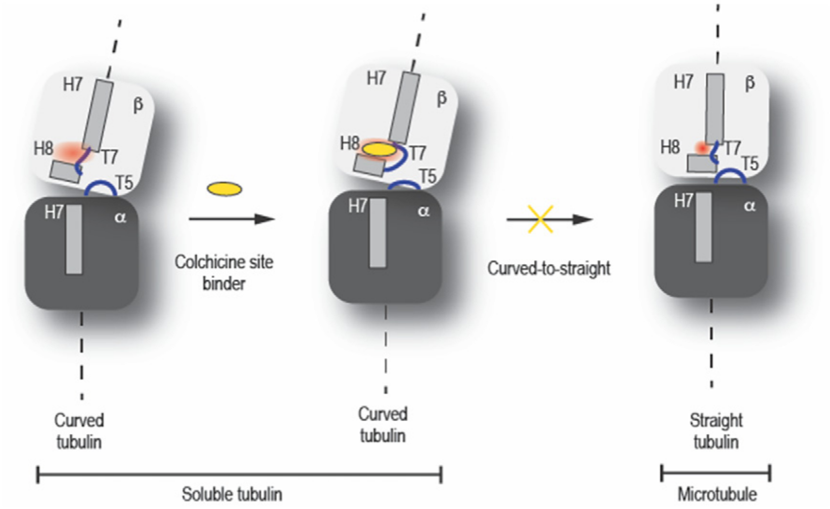

Figure EV3. Structural comparison and mechanism of action of colchicine-site ligands.

Schematic representation of the mechanism of action of colchicine-site ligands. (1) Soluble tubulin displays a characteristic curved conformation. (2) colchicine-site ligands (yellow sphere) block tubulin in the curved conformation by inhibiting the movement of helix βH7, βH8, βT7 loop, and αT5 loop thus preventing MT formation (3).

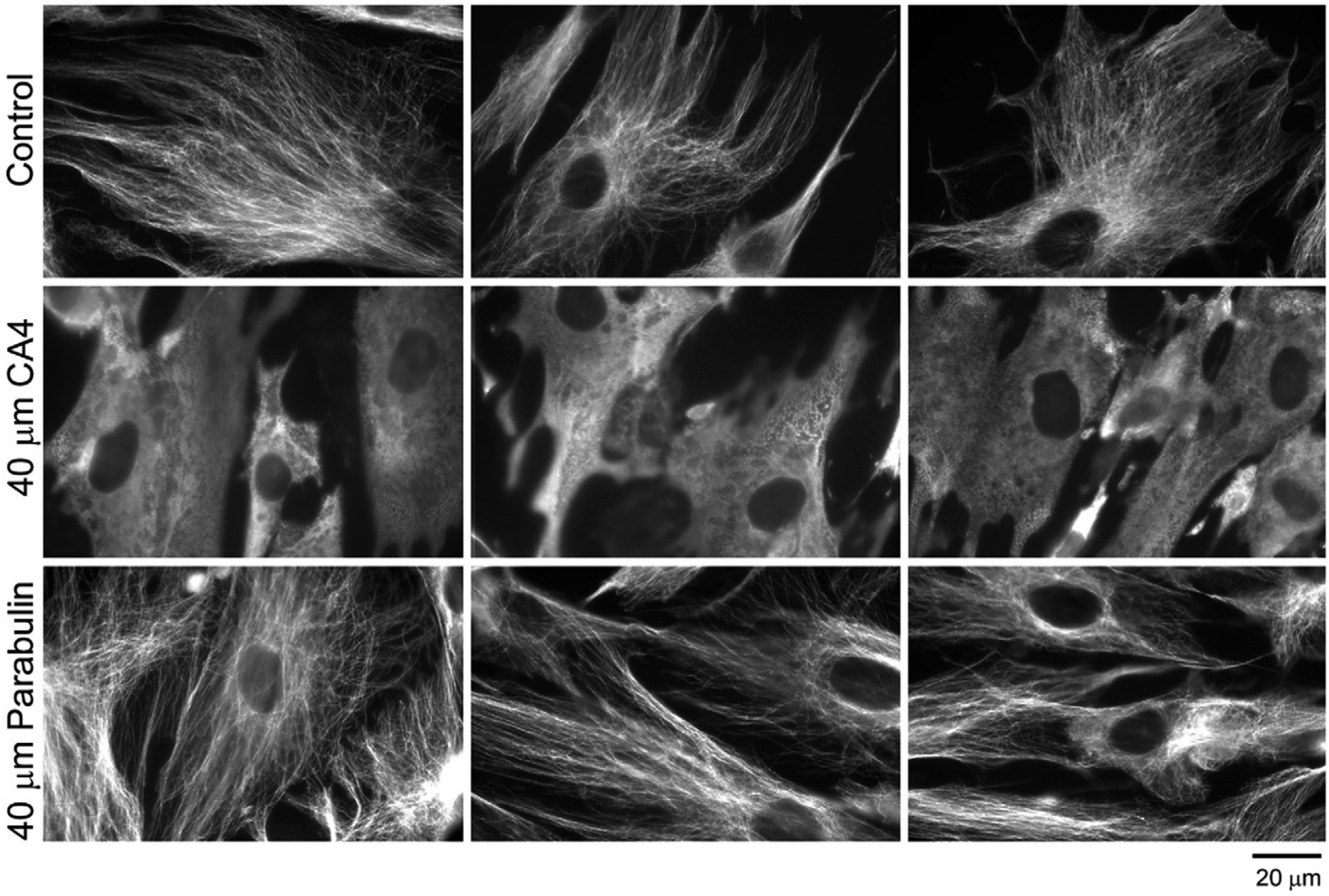

Figure EV4. Activity of CA4 and parabulin on vertebrate cell MTs.

Tubulin immunofluorescence showing the effect of 40 µM CA4 (middle row) and 40 µM parabulin (bottom row) compared to a null control (top row) on cultured human fibroblast cells. Both null control and parabulin samples have intact cytoplasmic MT arrays, whereas CA4 treatment completely disrupts MTs, leading to a diffuse distribution of free tubulin dimers.

© 2021 The Authors
