# Supplementary material for: Inhibiting parasite proliferation using a rationally designed anti‐tubulin agent
Source: EMBO Mol Med. 2021 Oct 18;13(11):e13818. doi: 10.15252/emmm.202013818 (PMC8573600; doi:10.15252/emmm.202013818)
Supplement: Supplementary file 3 [file EMMM-13-e13818-s002.pdf]

EMBO
Molecular Medicine

Inhibiting parasite proliferation using a rationally designed anti-tubulin agent

Natacha Gaillard, Ashwani Sharma, Izra Abbaali, Tianyang Liu, Fiona Shilliday, Alexander Cook, Valentin Ehrhard, Bangera Mamata, Anthony Roberts, Carolyn Moores, Naomi Morrissette, and Michel Steinmetz
DOI: 10.15252/emmm.202013818

Corresponding authors: Natacha Gaillard (natacha.gaillard@psi.ch) , Ashwani Sharma (ashwani.sharma@psi.ch)

Review Timeline:

Submission Date:	8th Jan 21
Editorial Decision:	1st Feb 21
Revision Received:	15th Jul 21
Editorial Decision:	4th Aug 21
Revision Received:	17th Sep 21
Accepted:	29th Sep 21

Editor: Lise Roth

Transaction Report:

(Note: With the exception of the correction of typographical or spelling errors that could be a source of ambiguity, letters and reports are not edited. Depending on transfer agreements, referee reports obtained elsewhere may or may not be included in this compilation. Referee reports are anonymous unless the Referee chooses to sign their reports.)

1st Feb 2021

Dear Dr. Gaillard,

Thank you for the full submission of your manuscript to EMBO Molecular Medicine following your presubmission inquiry. We have now received feedback from the three reviewers who agreed to evaluate your manuscript. As you will see from the reports below, the referees acknowledge the novelty and interest of the study, and are overall supporting publication of your work pending appropriate revisions.

Addressing the reviewers' concerns in full (see below for ref #1 point #1) will be necessary for further considering the manuscript in our journal, and acceptance of the manuscript will entail a second round of review.

After discussion within the editorial team and cross-commenting with the referees, we agree that solving the structure of a different human tubulin (ref #1, point #1) is beyond the scope of the current study, and therefore we would like you to discuss this point and potential limitations in your manuscript.
Referee #2 further indicated that the following points would help addressing this issue:
1. show that parabulin exhibits some specificity for the tetrahymena tubulin in vitro
2. show if there is decreased infectivity at parabulin concentrations that exhibit little toxicity to the mammalian cells.

EMBO Molecular Medicine encourages a single round of revision only and therefore, acceptance or rejection of the manuscript will depend on the completeness of your responses included in the next, final version of the manuscript. For this reason, and to save you from any frustrations in the end, I would strongly advise against returning an incomplete revision.

***

When submitting your revised manuscript, please carefully review the instructions that follow below. Failure to include requested items will delay the evaluation of your revision:

1) A .docx formatted version of the manuscript text (including legends for main figures, EV figures and tables). Please make sure that the changes are highlighted to be clearly visible.

2) Individual production quality figure files as .eps, .tif, .jpg (one file per figure).

3) A .docx formatted letter INCLUDING the reviewers' reports and your detailed point-by-point responses to their comments. As part of the EMBO Press transparent editorial process, the point-by-point response is part of the Review Process File (RPF), which will be published alongside your paper.

4) A complete author checklist, which you can download from our author guidelines (https://www.embopress.org/page/journal/17574684/authorguide#submissionofrevisions). Please insert information in the checklist that is also reflected in the manuscript. The completed author checklist will also be part of the RPF.

5) Before submitting your revision, primary datasets produced in this study need to be deposited in an appropriate public database (see https://www.embopress.org/page/journal/17574684/authorguide#dataavailability).
Please remember to provide a reviewer password if the datasets are not yet public.
The accession numbers and database should be listed in a formal "Data Availability " section (placed after Materials & Method).
Please note that the Data Availability Section is restricted to new primary data that are part of this study.

*** Note - All links should resolve to a page where the data can be accessed. ***

"This study includes no data deposited in external repositories"

6) We would also encourage you to include the source data for figure panels that show essential data. Numerical data should be provided as individual .xls or .csv files (including a tab describing the data). For blots or microscopy, uncropped images should be submitted (using a zip archive if multiple images need to be supplied for one panel). Additional information on source data and instruction on how to label the files are available at
.

7) Our journal encourages inclusion of *data citations in the reference list* to directly cite datasets that were re-used and obtained from public databases. Data citations in the article text are distinct from normal bibliographical citations and should directly link to the database records from which the data can be accessed. In the main text, data citations are formatted as follows: "Data ref: Smith et al, 2001" or "Data ref: NCBI Sequence Read Archive PRJNA342805, 2017". In the Reference list,

data citations must be labeled with "[DATASET]". A data reference must provide the database name, accession number/identifiers and a resolvable link to the landing page from which the data can be accessed at the end of the reference. Further instructions are available at .

8) We replaced Supplementary Information with Expanded View (EV) Figures and Tables that are collapsible/expandable online. A maximum of 5 EV Figures can be typeset. EV Figures should be cited as 'Figure EV1, Figure EV2" etc... in the text and their respective legends should be included in the main text after the legends of regular figures.

- For the figures that you do NOT wish to display as Expanded View figures, they should be bundled together with their legends in a single PDF file called *Appendix*, which should start with a short Table of Content. Appendix figures should be referred to in the main text as: "Appendix Figure S1, Appendix Figure S2" etc.

- Additional Tables/Datasets should be labeled and referred to as Table EV1, Dataset EV1, etc. Legends have to be provided in a separate tab in case of .xls files. Alternatively, the legend can be supplied as a separate text file (README) and zipped together with the Table/Dataset file.
See detailed instructions here:
.

9) The paper explained: EMBO Molecular Medicine articles are accompanied by a summary of the articles to emphasize the major findings in the paper and their medical implications for the non-specialist reader. Please provide a draft summary of your article highlighting
- the medical issue you are addressing,
- the results obtained and
- their clinical impact.

This may be edited to ensure that readers understand the significance and context of the research. Please refer to any of our published articles for an example.

10) For more information: There is space at the end of each article to list relevant web links for further consultation by our readers. Could you identify some relevant ones and provide such information as well? Some examples are patient associations, relevant databases, OMIM/proteins/genes links, author's websites, etc...

11) Author contributions: the contribution of every author must be detailed in a separate section (before the acknowledgments).

12) A Conflict of Interest statement should be provided in the main text

13) Every published paper now includes a 'Synopsis' to further enhance discoverability. Synopses are displayed on the journal webpage and are freely accessible to all readers. They include a short stand first (maximum of 300 characters, including space) as well as 2-5 one-sentences bullet points that summarizes the paper. Please write the bullet points to summarize the key NEW findings. They should be designed to be complementary to the abstract - i.e. not repeat the same text. We encourage inclusion of key acronyms and quantitative information (maximum of 30 words / bullet point). Please use the passive voice. Please attach these in a separate file or send them by email, we will incorporate them accordingly.

Please also suggest a striking image or visual abstract to illustrate your article. If you do please provide a png file 550 px-wide x 400-px high.

14) As part of the EMBO Publications transparent editorial process initiative (see our Editorial at http://embomolmed.embopress.org/content/2/9/329), EMBO Molecular Medicine will publish online a Review Process File (RPF) to accompany accepted manuscripts.
In the event of acceptance, this file will be published in conjunction with your paper and will include the anonymous referee reports, your point-by-point response and all pertinent correspondence relating to the manuscript. Let us know whether you agree with the publication of the RPF and as here, if you want to remove or not any figures from it prior to publication.
Please note that the Authors checklist will be published at the end of the RPF.

EMBO Molecular Medicine has a "scooping protection" policy, whereby similar findings that are published by others during review or revision are not a criterion for rejection. Should you decide to submit a revised version, I do ask that you get in touch after three months if you have not completed it, to update us on the status.

I look forward to receiving your revised manuscript.

Yours sincerely,

Lise Roth

Lise Roth, PhD
Editor
EMBO Molecular Medicine

***** Reviewer's comments *****

Referee #1 (Comments on Novelty/Model System for Author):

Adequacy of model system: See major concern 1 below

Referee #1 (Remarks for Author):

Tubulin, the building block of microtubules, is an attractive and well-described drug target, widely used in cancer therapies. In the present manuscript, the authors exploit structural differences between human and protozoa tubulins to design and test a parasite-specific drug.

This study is of general interest and warrants publication in EMBO Molecular Medicine. However, I have a three concerns and a few minor comments that shall be addressed prior to publication.

Major concerns:

(1) The entire rational of this study lies on the species-specific differences in parasite and human host tubulin. The authors beautifully solve the structure of T. thermophila αβ-tubulin in complex with DARPin1 and of paclitaxel-stabilized T. thermophila microtubules. For solving the human tubulin structure, the authors have chosen recombinant human α1β3-tubulin (Fig. 1F). For several reasons, I am not convinced that these are the best human isoforms. The authors should identify the human isoforms that are most similar to the parasite tubulin. Again with the rational that even the most similar human tubulins are different enough to not be affected by the drug. In particular β3-tubulin seems problematic as it is very distinct from all other ß-tubulin isoforms. In addition, ß3 is mostly present in neurons but not in actively dividing cells, which most likely are the ones affected most by a microtubule-targeting drug. It might be beyond the scope of this publication to solve the structure of a different human tubulin. However, the authors should at least identify the human isoforms most similar to T. thermophila tubulin, discuss the differences and the potential consequences for drug binding.

(2) The authors aim to identify an inhibitor, which is specific for parasite tubulin. In other words, identify an inhibitor that has no effect on human host microtubule dynamics. Unfortunately, the authors do not show any mammalian nor human microtubule growth in vitro in the presence of their parasite-specific inhibitor. This would be an essential control.

(3) The authors need to do a better job in providing figures that are fully comprehensive. For example, shouldn't there be a control for 4A? What are the concentrations used? These are not stated in the figure nor in the figure legend. Figure 4B: There is no scale bar in the lower part of the figure and nowhere is it stated what is labelled. Figure 4C: What is given, the mean? What are the error bars?

Minor points:
(1) General comment: It would be nice to see known, characterised microtubule dynamics (simply from brain tubulin) as a control and in comparison with the newly characterised T. thermophila tubulin (Figures 1C+D, 3 A-D, and S1).

(2) Figure 1E: While the text says that the microtubules have been stabilised with paclitaxel, the figure legend for 1E says "Cryo-electron microscopy density map of a 14 protofilament, GMPCPP-stabilized T. thermophila MT".

(3) Figure 2D: What is the control?

(4) Figures 3C: When was microtubule length measured? After the population reached steady state?

(5) The growth of microtubules in Figure S1 looks rather like unspecific polymerisation on coverslips that are not clean. There are very few and/or very short seeds. Is there another way of measuring the actual critical concentration of T. thermophila microtubules? Also, not sure screen shots (?) are the appropriate format to present original data.

(6) Figure S5B: What is the control?

(7) Figure S6A: What are the inhibitor concentrations?

Referee #2 (Comments on Novelty/Model System for Author):

The production of the cryo-EM structures of the Tetrahymena microtubules is a very nice result- these structures could be useful to a variety of labs that study alveolate tubulin, not just for drug design. The novelty is high because little work has been done on parasite tubulins, which potentially could be an excellent source of compounder for treating important diseases that are not commonly the focuse of drug design. This also goes to the medical impact. Using tetrahymena as a source of material was a good choice, considering the amount of material necessary to isolate tubulin. The use of toxoplasma for screening also allowed the authors to work with a bona fide parasite after doing the initial biophysical/structural work with tetrahymena.

Referee #2 (Remarks for Author):

Part 1 - Summary
In this work, the authors have employed structural biology and biophysics to identify compounds that bind to a novel binding site present in alveolate tubulins, which may serve as lead compounds for the discovery of antiparasitic drugs. The authors reasoned that understanding the structural differences between mammalian and parasite tubulin that account for differences in dinitroaniline sensitivity could be exploited for drug design. Like apicomplexan parasites, Tetrahymena thermophila tubulin is sensitive to dinitroaniline and therefore serves as an appropriate model for understanding structural differences between mammalian and parasite tubulin that account for dinitroaniline sensitivity.
The authors purified T. thermophila tubulin and showed that it assembles into microtubules as observed using cryo EM and undergoes dynamic instability in an in vitro TIRF assay with growth rates that are faster than those reported for bovine tubulin. A comparison of the crystalized structure of T. thermophila and mammalian tubulin bound to DARPin1 showed substantial differences in structure at the colchicine drug-binding site. To understand how these differences affect sensitivity of drugs that bind at this site, turbidity-based T. thermophila growth-inhibition assays were performed in the presence of colchicine, combretastatin A4 (CA4) and plinabulin which bind along different zones in the colchicine site. CA4 and plinabulin reduced cell growth however, CA4 was used as a basis for a rational drug design. One drug from a commercial ligand library, which the authors called parabulin, contains an altered aliphatic linker compared to CA4 and inhibited T. thermophila cell growth. Dynamic imaging of microtubules using an in vitro TIRF microscopy assay showed that parabulin reduced the average number of growth phases per microtubule seed from 0.15 to 0.03 per minute. However, there was little effect on the growth rate or length indicating that microtubule nucleation but not elongation is affected by parabulin.
Finally, the authors tested the efficacy of parabulin as an antiparasitic compound. The authors find few defects in host cell (fibroblast) growth in the presence of 25 µM of parabulin for 72 hours, which could result from reduced binding to mammalian tubulin relative to CA4. Treatment of parasite infected host cells with 40 µM parabulin for 48 hours resulted in decreased parasite replication. Treatment of parasite infected host cells with varying concentrations of parabulin for 7 days resulted in fewer plaques in a plaque assay.

Part 2 Major issues:
1. The authors should cite and discuss the work of the Wetzel lab (PMID: 32686409), who recently followed a similar

experimental path with leishmanial tubulin and screening of the Pathogen Box to identify a tubulin-binding antiparasitic drug. Interestingly, their compound appears to promote microtubule polymerization, unlike parabulin.
2. The authors never tested whether parabulin binds and alters the in vitro properties of mammalian tubulin. This is an important control that would directly test the hypothesis that parabulin targets T. thermophila and T. gondii tubulin but has diminished binding to mammalian tubulin.
3. Along these lines, the authors showed that parabulin treatment at 25 µM has essentially no effect on fibroblast viability, but performed their toxoplasma growth inhibition at 40 µM, which is much closer to a concentration that produced nearly 25% non-viable cells (per fig 4A). If parabulin affects mammalian cell viability, it is not difficult to see how sickly cells that are still viable could serve as poor hosts for toxoplasma, which would suppress the parasite's ability to proliferate. Put another way, there is no evidence that parabulin affects the parasite microtubules directly in cells. The authors could perform their infection experiments at a range of parabulin concentrations to address this- especially at concentrations closer to the amount that has no effect on mammalian cell viability. Is there a way they could directly look at the cortical MTs or spindle in Toxoplasma or tetrahymena to see if there are defects?
4. One could argue that immunofluorescence of parabulin-treated host cells showing an intact microtubule cytoskeleton is evidence that the drug does not bind to and destabilize mammalian cells, but microtubules are also visible in CA4 treated cells, which is a known microtubule destabilizer. In vitro data showing reduced effects on mammalian microtubule dynamics would directly test the hypothesis that parabulin targets parasite but not host cell tubulin.
5. In figure S6B, parabulin treated host cells look strikingly different from untreated controls, which raises concerns of host cell viability over the 7 days of the experiment in the presence of parabulin. Can the authors provide evidence that the host cells are viable over this entire course of this experiment? My concern is that reduced plaques may result from reduced host cell viability.

Part 3 Minor issues:
1. In the text the authors say "The average number of growth events per MT seed decreased from 0.15 +/- 0.001 to 0.3 +/- 0.03 per minute." Looking at the figure, I think this should read 0.03 per minute. If this is the case, the error is likely also different.
2. How was labeling of tubulin achieved with Alexa Fluor 488 carboxylic acid in the absence of a carbodiimide? Did the methods leave out this detail?
3. In figure 4, images of T gondii should be labeled to clarify how intracellular parasite number was scored.
4. The legend in Figure 4 should contain information about number of host cells counted and statistical differences comparing null, DMSO and parabulin-treated cells.
5. The author say that producing apicomplexan MTs has been unsuccessful- how is this known?
6. Can the authors clarify how they generated the thermophila MTs? Are the MTs used for cryo pure thermophila tubulin? The authors employed porcine seeds to polymerize the thermophila tubulin for the TIRF assays. This makes one wonder if you need those seeds to get the thermophila tubulin to polymerize.
7. The precise function of DARPin1 in the cryoEM work should be explained.
8. What is the degree of conservation between the Thermophila, Toxoplasma, and Plasmodium tubulins of the mutations present in the different MT drug binding sites?

Referee #3 (Comments on Novelty/Model System for Author):

This is the first high resolution structure of a protist tubulin to my knowledge ad its does by a world expert in tubulin strucutral biology. The resolution is excellent,

The target protein is taken from a convenient model protists but the compound is tested on the target pathogen. Also, the choice of model is well=justified

We need new treatments for neglected tropical diseases

Referee #3 (Remarks for Author):

This paper presents a really nice proof-of-concept for designing anti-parasite drug that target parasite tubulin but not human tubulin. Some of the best current drugs already target parasite tubulin, so it is a proven target, but their selectivity over human is unclear and their pharmacology is awful.

The tubulin structural biology is outstanding, as we might expect from a world leader. The interpretation of binding site chemistry is also outstanding.

The only mild negative in the paper is that the final parasite-selective compound is a bit weak in terms of IC50. This is acceptable for a proof-of-concept paper. The paper provides a clear roadmap for professional drug development including optimization of affinity and PK. That is a highly significant impact

To my knowledge, this paper is a first in rational design of a species-specific tubulin drug. That is a big deal, The abstract cøuld

spell it out more,

Minor concerns
Fig 4A provides a misleading impression of the potency of CA4, and therefore a misleading impression of the selectivity difference compared to the new compound. CA4 has an IC50 in the single digit nM range for blocking growth of human cells in culture. Fig 4A completely misses this IC50. This experiment should be re-done testing both drugs on a log scale and taking both (esp CA4) down below 1nM. We should be able to see the IC50 for CA4 around 3nM and be able to compare to compare to the lack of an IC50 for the new compound. The selectivity must be at least 3,000-fold, but Fig 4A fails to capture this point. We need to see IC50 values for both drugs to make this central point properly,

Fig 4C is also not very professional in terms of dose rage. It would be good to re-do this experiment again using a log scale of drug concentration so we can see a clear inflection point for the new compound, and thus a reliable IC50 value. This is less important than Fig 4A, but still it would be much better to use a log scale for the drug and go to lower values so we can see a clear IC50 inflection point.

Point by point answer to the editor and referees

Editor:

After discussion within the editorial team and cross-commenting with the referees, we agree that solving the structure of a different human tubulin (ref #1, point #1) is beyond the scope of the current study, and therefore we would like you to discuss this point and potential limitations in your manuscript.

Response: We are thankful that the editorial team agreed that solving additional structures of different human tubulins is beyond the scope of the current study. We even think that it does not make much sense since the residue composition of the colchicine site of human tubulins is invariant amongst human tubulin isoforms. This is highlighted by our newly performed multiple sequence alignments with all human alpha- and beta-tubulin sequences (new Expended figures EV5 and EV6), which revealed that the critical residues involved in specific colchicine-site ligand binding, i.e., Ala250 and Ala316 in protozoa beta tubulins and Ser250 and Ser 316 in human beta tubulins, are invariant within the two phyla. These observations are now mentioned in the revised manuscript on page 6, line 11. See also our response to Referee 1 Major Point 1.

Referee #2 further indicated that the following points would help addressing this issue:

1. show that parabulin exhibits some specificity for the tetrahymena tubulin in vitro

Response: In order to confirm that parabulin is specific for tetrahymena tubulin we performed in vitro microtubule dynamics TIRF assays with porcine brain tubulin in the presence of increasing amounts of parabulin. We measured the growth rates, the growth lengths and the growth event numbers for the DMSO control and 5 different concentrations of parabulin from 0.1 µM up to 100 µM. Parabulin shows no significant effect on the growth dynamics of porcine microtubules highlighting a specific inhibitory effect on tetrahymena microtubules. These results are now described on page 8, line 9 of the revised manuscript and are shown in expended figure EV1.

2. show if there is decreased infectivity at parabulin concentrations that exhibit little toxicity to the mammalian cells.

Response: We have several lines of evidence that suggest that parabulin affects parasite replication directly and that reduced replication is not simply due to host cell toxicity compromising parasite growth:

1. *Serial passage of parasites in parabulin causes aberrant forms that are consistent with microtubule defects that disconnect coordinated nuclear division and daughter cell budding. This data is now represented in Figure 4D. We have added text to the figure legend and the revised manuscript to refer to this information (page 9, line 18).*
2. *Infectivity refers to parasite invasion. Parabulin affects both intracellular replication (Figure 4B and host cell invasion (now included in the supplemental data). The plaque assay used to measure the EC50 value captures both invasion and intracellular replication because plaques arise after several rounds of invasion, replication, and parasite egress (resulting in host cell lysis). We have added an invasion assay to the supplemental data (expended figure EV8B).*
3. *We show MTT assays for sub-confluent (replicating) and confluent (contact inhibited) HFF cells. After 3 days of treatment, 40 µM parabulin reduces viability of confluent cells to 76% of controls. This information is now mentioned page 8, line 23 and shown in the supplemental data Expended Figure EV8A.*

Referee #1 Major Points:

(1) The entire rational of this study lies on the species-specific differences in parasite and human host tubulin. The authors beautifully solve the structure of T. thermophila αβ-tubulin in complex with DARPin1 and of paclitaxel-stabilized T. thermophila microtubules. For solving the human tubulin structure, the authors have chosen recombinant human α1β3-tubulin (Fig. 1F). For several reasons, I am not convinced that these are the best human isoforms. The authors should identify the human isoforms that are most similar to the parasite tubulin. Again with the rational that even the most similar human tubulins are different enough to not be affected by the drug. In particular β3-tubulin seems problematic as it is very distinct from all other ß-tubulin isoforms. In addition, β3 is mostly present in neurons but not in actively dividing cells, which most likely are the ones affected most by a microtubule-targeting drug. It might be beyond the scope of this publication to solve the structure of a different human tubulin. However, the authors should at least identify the human isoforms most similar to T. thermophila tubulin, discuss the differences and the potential consequences for drug binding.

Response: The aim of the study was to demarcate and exploit subtle structural differences of tubulin drug-binding sites for parasite specific inhibitor design. Although, there are sequence differences among the different human tubulin isoforms (new expended figure EV5 and EV6), these are mostly located at the C-terminal tails of both tubulin isoforms and rarely at the drug-binding sites. Furthermore, the particular sequence differences we highlighted in this study between protozoa and human tubulins are well conserved among human tubulin isoforms. We have now included a multiple sequence alignment of all human alpha and all human beta tubulins to compare drug-binding site residues with the ones from Toxoplasma, Plasmodium and Tetrahymena tubulins (expended figures EV5 and EV6). Notably, the critical residue differences between human and protozoa tubulins in the colchicine-binding site, which we exploited in this study for specific inhibitor design (i.e., Ala250 and Ala316, and Ser250 and Ser 316 in human and protozoa tubulins, respectively) are conserved in all human tubulin isoforms. These observations are now mentioned in the revised manuscript on page 6, line 11.
Notably, we used beta*3 tubulin for structural studies because of two main reasons: 1)* alpha*1/*beta*3 tubulin is the only tubulin isoform that we were able to express recombinantly in sufficient amounts for structural studies, and 2) structural data on mammalian alpha1/beta2 is already published (Steinmetz MO and Prota AE. Trends Cell Biol. 2018 Oct;28(10):776-792. doi: 10.1016/j.tcb.2018.05.001 PMID: 29871823 ; Ti SC, et al. Dev Cell. 2018 Oct 22;47(2):175-190.e5. doi: 10.1016/j.devcel.2018.08.014, PMID: 30245156.).*

(2) The authors aim to identify an inhibitor, which is specific for parasite tubulin. In other words, identify an inhibitor that has no effect on human host microtubule dynamics. Unfortunately, the authors do not show any mammalian nor human microtubule growth in vitro in the presence of their parasite-specific inhibitor. This would be an essential control.

Response: We now measured growth rates growth lengths and growth event numbers of porcine brain microtubules for DMSO control and 5 different concentrations of parabulin from 0.1 µM up to 100 µM. Parabulin shows no significant effect on growth dynamics of porcine tubulin highlighting a specific inhibitory effect on tetrahymena microtubules. These data have been added as an expended figure EV1D and are mentioned on page 8, line 10 of the revised manuscript.

(3) The authors need to do a better job in providing figures that are fully comprehensive. For example, shouldn't there be a control for 4A? What are the concentrations used? These are not stated in the figure nor in the figure legend. Figure 4B: There is no scale bar in the lower part of the figure and nowhere is it stated what is labelled. Figure 4C: What is given, the mean? What are the error bars?

<u>Response</u>*: The absorbance measurements for all treatment conditions in the MTT assay are normalized to the null control condition that is set to 100% growth. We have modified the figure and the figure legend to make this clearer. We now include a scale bar to the image of GFP-expressing intracellular parasites. The error bars represent standard error of the mean. We have now revised the figure legend to encompass this information. We also added a new panel to this figure to illustrate how serial passage of parasites in parabulin causes aberrant forms that are consistent with microtubule defects that disconnect nuclear division and daughter cell budding. This data is now represented in an additional panel in Figure 4 and we have amended the figure legend to encompass this data.*

<u>Referee #1 Minor points:</u>

(1) General comment: It would be nice to see known, characterized microtubule dynamics (simply from brain tubulin) as a control and in comparison with the newly characterized T. thermophila tubulin (Figures 1C+D, 3 A-D, and S1).

<u>Response</u>*: The data for the porcine brain microtubules have been added to the revised manuscript (page 8, line 10) and the growth rates of T. thermophila microtubules were compared to B. taurus and C. elegans microtubules and is now reported in the revised manuscript on page 5, line 1.*

(2) Figure 1E: While the text says that the microtubules have been stabilized with paclitaxel, the figure legend for 1E says "Cryo-electron microscopy density map of a 14 protofilament, GMPCPP-stabilized T. thermophila MT".

<u>Response</u>*: This discrepancy has been clarified in the revised manuscript.*

(3) Figure 2D: What is the control?

<u>Response</u>*: The control in this experiment is DMSO. It has been clarified in the figure legend.*

(4) Figures 3C: When was microtubule length measured? After the population reached steady state?

<u>Response</u>*: : MT length was measured on the dynamic microtubules once they had reached steady state growth. This information has been added to the figure legend.*

(5) The growth of microtubules in Figure S1 looks rather like unspecific polymerisation on coverslips that are not clean. There are very few and/or very short seeds. Is there another way of measuring the actual critical concentration of T. thermophila microtubules? Also, not sure screen shots (?) are the appropriate format to present original data.

<u>Response</u>*: The Morrissette lab has previously measured the Tetrahymena tubulin critical concentration in the absence of seeds* (Lyons-Abbott S. *et al.* Eukaryot Cell. 2010 Dec;9(12):1825-34 PMID: 20870876*). In*

the reported assays, wild-type Tetrahymena tubulin has a Cc value of 0.55 ± 0.09 mg/ml (5.5 µM). The images included in expended figure EV1 support as well our statement concerning the very high levels of polymerization by tetrahymena tubulin at concentrations more typically used for mammalian brain tubulin in vitro.

(6) Figure S5B: What is the control?

Response: *The control in this experiment is DMSO. It has been clarified in the figure legend.*

(7) Figure S6A: What are the inhibitor concentrations?

Response: *Both compounds were used at 40 µM. It has been clarified in the figure legend. Please note that Figure S6 is now expended figure EV8 in the resubmitted manuscript.*

Referee #2 Major issues:

1. The authors should cite and discuss the work of the Wetzel lab (PMID: 32686409), who recently followed a similar experimental path with leishmanial tubulin and screening of the Pathogen Box to identify a tubulin-binding antiparasitic drug. Interestingly, their compound appears to promote microtubule polymerization, unlike parabulin.

Response: *We now refer to this paper in the Discussion section of our revised manuscript (page 10, line 7).*

2. The authors never tested whether parabulin binds and alters the in vitro properties of mammalian tubulin. This is an important control that would directly test the hypothesis that parabulin targets T. thermophila and T. gondii tubulin but has diminished binding to mammalian tubulin.

Response: *In order to confirm that parabulin is specific for tetrahymena tubulin, we performed in vitro microtubule dynamics TIRF assays with porcine brain tubulin in the presence of increasing amounts of parabulin. We measured the growth rates, the growth lengths and the growth event numbers for the DMSO control and 5 different concentrations of parabulin from 0.1 µM up to 100 µM. Parabulin shows no significant effect on the growth dynamics of porcine microtubules highlighting a specific inhibitory effect on tetrahymena microtubules. These results are described on page 8, line 10 of the revised manuscript and are shown in expended figure EV1D.*

3. Along these lines, the authors showed that parabulin treatment at 25 µM has essentially no effect on fibroblast viability, but performed their toxoplasma growth inhibition at 40 µM, which is much closer to a concentration that produced nearly 25% non-viable cells (per Fig. 4A). If parabulin affects mammalian cell viability, it is not difficult to see how sickly cells that are still viable could serve as poor hosts for toxoplasma, which would suppress the parasite's ability to proliferate. Put another way, there is no evidence that parabulin affects the parasite microtubules directly in cells. The authors could perform their infection experiments at a range of parabulin concentrations to address this- especially at concentrations closer to the amount that has no effect on mammalian cell viability. Is there a way they could directly look at the cortical MTs or spindle in Toxoplasma or tetrahymena to see if there are defects?

Response: *We agree that the parasite growth assays could be influenced by host cell toxicity. This is the complication and challenge of searching for drug targets and selective inhibitors for intracellular eukaryotic pathogens. The parasite growth assays were done in confluent (contact inhibited, non-dividing) primary human foreskin fibroblast (HFF) cells. We now provide MTT data for both subconfluent and confluent HFF cell cultures in a range from 10 to 40 μM parabulin in a supplemental figure (expended figure EV8A). The data that parabulin slows replication of the related free-living protozoan Tetrahymena is also consistent with direct action on parasite functions (Fig. 2C).*

With respect to visualizing microtubules, in preliminary experiments we used GFP tagged markers of spindle and subpellicular microtubules in replicating parasites to visualize overt defects in microtubule population but we do not see significant changes. We suspect that parabulin slows assembly of a functional spindle and therefore delays nuclear division by acting as a microtubule poison at the plus ends, much like sub-stoichiometric amounts of colchicine do (Margolis RL et al. Proc Natl Acad Sci U S A. 1977 Aug;74(8):3466-70 PMID: 269406). It is worth noting that Toxoplasma undergoes a closed mitosis and since their chromosomes remain durably attached to specialized patches of the nuclear envelope, spindle microtubules solely function to mechanically separate the chromosome sets for nuclear division. Parasites serially passaged in 40-80 μM oryzalin acquire replication defects that are consistent with nuclear division delay disconnected from cytokinesis. This is now mentioned in the text page 9, line 18, and the images are added to Figure 4 and described in the figure legend.

4. One could argue that immunofluorescence of parabulin-treated host cells showing an intact microtubule cytoskeleton is evidence that the drug does not bind to and destabilize mammalian cells, but microtubules are also visible in CA4 treated cells, which is a known microtubule destabilizer. In vitro data showing reduced effects on mammalian microtubule dynamics would directly test the hypothesis that parabulin targets parasite but not host cell tubulin.

Response: *We now show new tubulin fluorescence images using a new batch of CA4 and provide multiple panels for each condition. Microtubule disruption in the presence of CA4 is complete and Parabulin looks similar to null control samples.*

5. In figure S8B, parabulin treated host cells look strikingly different from untreated controls, which raises concerns of host cell viability over the 7 days of the experiment in the presence of parabulin. Can the authors provide evidence that the host cells are viable over this entire course of this experiment? My concern is that reduced plaques may result from reduced host cell viability.

Response: *We agree that the parasite growth assays could be influenced by host cell toxicity. This is the complication and challenge of searching for drug targets and selective inhibitors for intracellular eukaryotic pathogens. We now provide MTT data for confluent HFF cells in a range from 10 to 40 μM in a supplemental figure (expended figure EV8A).*

Referee #2 Minor issues:

1. In the text the authors say "The average number of growth events per MT seed decreased from 0.15 +/- 0.001 to 0.3 +/- 0.03 per minute." Looking at the figure, I think this should read 0.03 per minute. If this is the case, the error is likely also different.

Response: The reviewer is correct that this was a typo, and we have now corrected the text accordingly.

2. How was labeling of tubulin achieved with Alexa Fluor 488 carboxylic acid in the absence of a carbodiimide? Did the methods leave out this detail?

Response: The labelling with Alexa Fluor 488 was done in our laboratory as described in Castoldi M. et al. Protein Expr Purif. 2003 Nov;32(1):83-8 PMID: 14680943. The detailed protocol can be found in our Materials and Methods section on page 2, line 27.

3. In figure 4, images of T gondii should be labeled to clarify how intracellular parasite number was scored.

Response: Thank you for noticing this omission. The parasite numbers are now labeled.

4. The legend in Figure 4 should contain information about number of host cells counted and statistical differences comparing null, DMSO and parabulin-treated cells.

Response: GFP-expressing Toxoplasma tachyzoites were added to HFF monolayers in MatTek coverslip dishes to allow invasion for 30 minutes before all dishes were washed to remove extracellular parasites. Warm (37 °C) control or drug-containing media was added to dishes. From this point on, we imaged these samples every 12 hours and collected 10 random fields of view for subsequent quantification. For each image, we enumerated numbers of parasites in individual vacuoles per field. Since it is not always accurate to quantify parasite numbers beyond 8 parasites per vacuole, we scored vacuoles as containing 1, 2, 4 or 8+ parasites. Beyond 36 hours, untreated parasites begin to lyse out of cells and begin a second cycle of replication, so the 36 hour point is an ideal time to compare control and treated samples. We now show three biological replicates with values normalized to 100% for each condition and each replicate.

5. The authors say that producing apicomplexan MTs has been unsuccessful how is this known?

Response: We cloned Toxoplasma alpha and beta tubulins for recombinant expression in insect cells using a similar strategy as used for the human tubulin. However, we failed to express and purify any Toxoplasma tubulin using this methodology. Isolation of native tubulin in sufficient amounts for structural studies from Toxoplasma is very difficult due to technical reasons. Toxoplasma is an obligate intracellular parasite that only replicates inside cultured vertebrate cells grown in fetal bovine serum.

Their microtubule cytoskeleton consists of 22 ~5 µm long subpellicular microtubules and a spindle consisting of 28 ~1.5 µm long microtubules. An individual T. gondii tachyzoite has a tubulin concentration of approximately 0.073 pg/cell. Tetrahymena, a free-living relative has 30 pg tubulin/cell (Lyons-Abbott et al. Eucaryot Cell, 2010 Dec; 9(12): 1825-1834, PMID: 20870876) -- over 400 times more tubulin per cell than Toxoplasma and can be grown in inexpensive media. It is therefore prohibitively expensive and inefficient to purify T. gondii tubulin from tachyzoites and Tetrahymena is an inexpensive substitute.

6. Can the authors clarify how they generated the thermophila MTs? Are the MTs used for cryo pure thermophila tubulin? The authors employed porcine seeds to polymerize the thermophila tubulin for the TIRF assays. This makes one wonder if you need those seeds to get the thermophila tubulin to polymerize.

Response: The pure T. thermophila microtubules were polymerized in the presence of GTP and stabilized with Taxol for the cryo-EM work (see Materials and Methods). The porcine microtubule seeds used in the TIRF microscopy assay encompass biotinylated tubulin in order to anchor the microtubules to the cover slides for our dynamics experiments. Dynamic T. thermophila microtubules are nucleated from the seeds from which we measured microtubule dynamics parameters (Fig. 3).

The precise function of DARPin1 in the cryoEM work should be explained.

Response: The DARPin1 was only used as a crystallization tool to obtain well diffracting crystals of human and tetrahymena tubulin. It was not used in our cryo-EM work.

7. What is the degree of conservation between the Thermophila, Toxoplasma, and Plasmodium tubulins of the mutations present in the different MT drug binding sites?

Response: The different drug-binding site of Thermophila, Toxoplasma and Plasmodium are extremely similar as highlighted in the alignment expended figures EV2 and EV3.

Referee #3 (Comments on Novelty/Model System for Author):

1. To my knowledge, this paper is a first in rational design of a species-specific tubulin drug. That is a big deal, The abstract could spell it out more.

Response: We thank this referee for this comment and we have highlighted it in our abstract.

2. Fig 4A provides a misleading impression of the potency of CA4, and therefore a misleading impression of the selectivity difference compared to the new compound. CA4 has an IC50 in the single digit nM range for blocking growth of human cells in culture. Fig 4A completely misses this IC50. This experiment should be re-done testing both drugs on a log scale and taking both (esp CA4) down below 1nM. We should be able to see the IC50 for CA4 around 3nM and be able to compare to compare to the lack of an IC50 for the new compound. The selectivity must be at least 3,000-fold, but Fig 4A fails to capture this point. We need to see IC50 values for both drugs to make this central point properly.

Response: The fibroblast toxicity assay shown in Figure 4A was not designed to be an IC50 assay for CA4. The CA4 values are shown in comparison to comparable parabulin concentrations. We used primary

human fibroblasts plated to be subconfluent so that we could visualize any compound effects on proliferating cells. In the range of concentrations shown, CA4 inhibits cell division but does not kill the previously adherent cells. This is why the reduction to 50% is seen from 25 to 100 µM CA4.

3. Fig 4C is also not very professional in terms of dose rage. It would be good to re-do this experiment again using a log scale of drug concentration so we can see a clear inflection point for the new compound, and thus a reliable IC50 value. This is less important than Fig 4A, but still it would be much better to use a log scale for the drug and go to lower values so we can see a clear IC50 inflection point.

Response: *This is the standard way to assess compound efficacy on Toxoplasma tachyzoite growth (for example, see papers by Pfefferkorn ER. et al. Antimicrob Agents Chemother. 1992 May;36(5):1091-6 PMID: 1510399; D'Angelo JG et al. J Antimicrob Chemother. 2009 Jan;63(1):146-50 PMID: 18988681 and our previous work on dinitroaniline compounds (Ma C. Mol Biol Cell. 2007 Dec;18(12):4711-20. PMID: 17881728). It captures effects of drugs that act on the first replication cycle (the dinitroanilines, Parabulin) as well as drugs that have a delayed (cumulative) effect that requires several rounds of host cell invasion and replication before becoming apparent (e.g,. clindamycin).*

4th Aug 2021

Dear Dr. Gaillard,

Thank you for the submission of your revised manuscript to EMBO Molecular Medicine. We have now received the enclosed reports from two of the referees who had reviewed your original manuscript. As you will see, they are now supportive of publication, and I am therefore pleased to inform you that we will be able to accept your manuscript once the following points will be addressed:

1/ Referees' comments:
Please address the remaining minor concerns from the referees.

2/ Main manuscript text:
- Please address the queries from our data editors in track changes mode in the main manuscript file labelled 'Data edited MS file. Please use this file for any further modification.
- Please remove the yellow highlights and only keep in track changes the new modifications.
- Please provide up to 5 keywords.
- Please reorder the manuscript as follows: Introduction, Results and Discussion, Material and Methods, Data Availability, Acknowledgements, Author Contributions, Conflict of Interest and References.
- Material and Methods: We note that your Material and Methods are in the Appendix. Please place it in the main manuscript file. When referring to previously published methods, please make sure that the information is easily accessible to the public.
o Cells: please indicate the origin of the cells
o Invasion assay: please indicate the antibody dilution
- Please rename the Data Availability section and provide an URL to access the data. Please note that the data must be public before acceptance of the manuscript.
- Acknowledgements: Please add Medical Research Council, UK in the submission system.
- Author contributions: the contribution of every author must be detailed in a separate section. Please add Mamata Bangera in the submission system.
- A Conflict of Interest statement should be provided in the main text. The patent should be listed in this section. Please note that I could not access the patent.
- The References should be renamed, and the references should be listed in alphabetical order, with 10 authors before et al.

3/ Figures and Appendix:
- A maximum of 5 Expanded View (EV) Figures can be typeset. EV Figures should be cited as 'Figure EV1, Figure EV2" etc... in the text and their respective legends should be included in the main text after the legends of regular figures. The additional figures should be bundled together with their legends in a single PDF file called *Appendix*, which should start with a short Table of Content. Appendix figures should be referred to in the main text as: "Appendix Figure S1, Appendix Figure S2" etc.
- Tables EV1 and EV2 need titles/legends added to the files.
- Please carefully check that all figures and figure panels are referenced in the main text (figure callouts are missing for Fig 2D; Fig 3D; Fig 4D; Figure EV10).

4) We would also encourage you to include the source data for figure panels that show essential data. Numerical data should be provided as individual .xls or .csv files (including a tab describing the data). For blots or microscopy, uncropped images should be submitted (using a zip archive if multiple images need to be supplied for one panel). Additional information on source data and instruction on how to label the files are available at
.

5) Checklist:
Please complete the statistic information in section B.
Please indicate in section C/6 the references of the antibodies used in the study. Please indicate in C/7 whether the cells were tested for mycoplasma contamination and authenticated.
Please provide in section F/19 the URL to access the deposited data.

6) The paper explained: EMBO Molecular Medicine articles are accompanied by a summary of the articles to emphasize the major findings in the paper and their medical implications for the non-specialist reader. Please provide a draft summary of your article highlighting
- the medical issue you are addressing,
- the results obtained and
- their clinical impact.
This may be edited to ensure that readers understand the significance and context of the research. Please refer to any of our published articles for an example.

7) Every published paper now includes a 'Synopsis' to further enhance discoverability. Synopses are displayed on the journal webpage and are freely accessible to all readers. They include a short stand first (maximum of 300 characters, including space) as well as 2-5 one-sentences bullet points that summarizes the paper. Please write the bullet points to summarize the key NEW findings. They should be designed to be complementary to the abstract - i.e. not repeat the same text. We encourage inclusion of key acronyms and quantitative information (maximum of 30 words / bullet point). Please use the passive voice. Please attach these in a separate file or send them by email, we will incorporate them accordingly.

Please also suggest a striking image or visual abstract to illustrate your article as a PNG file 550 px wide x 300-600 px high.

8) As part of the EMBO Publications transparent editorial process initiative (see our Editorial at http://embomolmed.embopress.org/content/2/9/329), EMBO Molecular Medicine will publish online a Review Process File (RPF) to accompany accepted manuscripts.
This file will be published in conjunction with your paper and will include the anonymous referee reports, your point-by-point response and all pertinent correspondence relating to the manuscript. Let us know whether you agree with the publication of the RPF and as here, if you want to remove or not any figures from it prior to publication.
Please note that the Authors checklist will be published at the end of the RPF.

I look forward to receiving your revised manuscript.

With kind regards,

Lise Roth

Lise Roth, PhD
Editor
EMBO Molecular Medicine

***** Reviewer's comments *****

Referee #1 (Remarks for Author):

The most important control has been added: Effect of parabulin on mammalian microtubules (EV1). However, it is important to add the tubulin conc. used in these ctrl experiments, otherwise the measured parameters of microtubule dynamics (such as polymerisation velocity) can not be compared to published data.

Mechanistically, it is interesting that the average number of growth events per MT seed decreased while no significant differences in growth velocity is measured. Can the authors briefly discuss!

Referee #2 (Comments on Novelty/Model System for Author):

The authors describe a new lead compound that has potential as a tubulin-binding antiparasitic. The employed a wide-range of techniques including crystallography and cell biology to characterize this compound. It could become a starting point for the discovery of novel drugs for neglected tropical diseases, which are sorely needed.

Referee #2 (Remarks for Author):

The authors have done a good job addressing my criticisms. I have one small comment- on page 10, line 6-8, they claim that "parasites develop resistance quickly after introduction of new therapies..". While this is true in many cases, such as malaria, it is not universal. For example, Toxoplasma gondii, which is an important part of this work, has not developed resistance to its treatments. This has a lot to do with the mode of transmission of Toxoplasma in humans, but perhaps it would be worth providing a more qualified statement.

The authors performed the requested editorial changes.

29th Sep 2021

Dear Dr. Gaillard,

Thank you for submitting the last revised files. I am pleased to inform you that your manuscript is accepted for publication and is now being sent to our publisher to be included in the next available issue of EMBO Molecular Medicine.

Please read below for additional IMPORTANT information regarding your article, its publication and the production process.

Congratulations on your interesting work!

With kind regards,

Lise Roth

Lise Roth, Ph.D
Editor
EMBO Molecular Medicine

Follow us on Twitter @EmboMolMed

EMBO PRESS

YOU MUST COMPLETE ALL CELLS WITH A PINK BACKGROUND ↓

PLEASE NOTE THAT THIS CHECKLIST WILL BE PUBLISHED ALONGSIDE YOUR PAPER

Corresponding Author Name: Natacha Gaillard and Ashwani Sharma
Journal Submitted to: EMBO Molecular Medicine
Manuscript Number: EMM-2020-13818

Reporting Checklist For Life Sciences Articles (Rev. June 2017)

This checklist is used to ensure good reporting standards and to improve the reproducibility of published results. These guidelines are consistent with the Principles and Guidelines for Reporting Preclinical Research issued by the NIH in 2014. Please follow the journal's authorship guidelines in preparing your manuscript.

A- Figures

1. Data

The data shown in figures should satisfy the following conditions:

- the data were obtained and processed according to the field's best practice and are presented to reflect the results of the experiments in an accurate and unbiased manner.
- figure panels include only data points, measurements or observations that can be compared to each other in a scientifically meaningful way.
- graphs include clearly labeled error bars for independent experiments and sample sizes. Unless justified, error bars should not be shown for technical replicates.
- if n< 5, the individual data points from each experiment should be plotted and any statistical test employed should be justified
- Source Data should be included to report the data underlying graphs. Please follow the guidelines set out in the author ship guidelines on Data Presentation.

2. Captions

Each figure caption should contain the following information, for each panel where they are relevant:

- a specification of the experimental system investigated (eg cell line, species name).
- the assay(s) and method(s) used to carry out the reported observations and measurements
- an explicit mention of the biological and chemical entity(ies) that are being measured.
- an explicit mention of the biological and chemical entity(ies) that are altered/varied/perturbed in a controlled manner.
- the exact sample size (n) for each experimental group/condition, given as a number, not a range;
- a description of the sample collection allowing the reader to understand whether the samples represent technical or biological replicates (including how many animals, litters, cultures, etc.).
- a statement of how many times the experiment shown was independently replicated in the laboratory.
- definitions of statistical methods and measures:
  - common tests, such as t-test (please specify whether paired vs. unpaired), simple χ^2 tests, Wilcoxon and Mann-Whitney tests, can be unambiguously identified by name only, but more complex techniques should be described in the methods section;
  - are tests one-sided or two-sided?
  - are there adjustments for multiple comparisons?
  - exact statistical test results, e.g., P values = x but not P values < x;
  - definition of 'center values' as median or average;
  - definition of error bars as s.d. or s.e.m.

Any descriptions too long for the figure legend should be included in the methods section and/or with the source data.

In the pink boxes below, please ensure that the answers to the following questions are reported in the manuscript itself. Every question should be answered. If the question is not relevant to your research, please write NA (non applicable). We encourage you to include a specific subsection in the methods section for statistics, reagents, animal models and human subjects.

USEFUL LINKS FOR COMPLETING THIS FORM

http://www.antibodypedia.com
http://1degreebio.org
http://www.equator-network.org/reporting-guidelines/improving-bioscience-research-repor
http://grants.nih.gov/grants/olaw/olaw.htm
http://www.mrc.ac.uk/Ourresearch/Ethicsresearchguidance/Useofanimals/index.htm
http://ClinicalTrials.gov
http://www.consort-statement.org
http://www.consort-statement.org/checklists/view/32-consort/66-title
http://www.equator-network.org/reporting-guidelines/reporting-recommendations-for-tumo
http://datadryad.org
http://figshare.com
http://www.ncbi.nlm.nih.gov/gap
http://www.ebi.ac.uk/ega
http://biomodels.net/
http://biomodels.net/miriam/
http://jjj.biochem.sun.ac.za
http://oba.od.nih.gov/biosecurity/biosecurity_documents.html
http://www.selectagents.gov/

B- Statistics and general methods

Please fill out these boxes ↓ (Do not worry if you cannot see all your text once you press return)

1.a. How was the sample size chosen to ensure adequate power to detect a pre-specified effect size?	NA
1.b. For animal studies, include a statement about sample size estimate even if no statistical methods were used.	NA
2. Describe inclusion/exclusion criteria if samples or animals were excluded from the analysis. Were the criteria pre-established?	NA
3. Were any steps taken to minimize the effects of subjective bias when allocating animals/samples to treatment (e.g. randomization procedure)? If yes, please describe.	NA
For animal studies, include a statement about randomization even if no randomization was used.	NA
4.a. Were any steps taken to minimize the effects of subjective bias during group allocation or/and when assessing results (e.g. blinding of the investigator)? If yes please describe.	NA
4.b. For animal studies, include a statement about blinding even if no blinding was done	NA
5. For every figure, are statistical tests justified as appropriate?	Yes
Do the data meet the assumptions of the tests (e.g., normal distribution)? Describe any methods used to assess it.	NA
Is there an estimate of variation within each group of data?	Yes

Is the variance similar between the groups that are being statistically compared?	NA

C- Reagents

6. To show that antibodies were profiled for use in the system under study (assay and species), provide a citation, catalog number and/or clone number, supplementary information or reference to an antibody validation profile. e.g., Antibodypedia (see link list at top right), 1DegreeBio (see link list at top right).	Samples were stained with a 1:1000 anti-SAG1 mouse monoclonal antibody (Thermofisher MA5-18268) and detected with a 1:1000 dilution of a goat anti-mouse Alexa 594 secondary antibody (Thermofisher A-11005).
7. Identify the source of cell lines and report if they were recently authenticated (e.g., by STR profiling) and tested for mycoplasma contamination.	Cell lines used reported in our Manuscript. Tested for mycoplasma contamination.

* for all hyperlinks, please see the table at the top right of the document

D- Animal Models

8. Report species, strain, gender, age of animals and genetic modification status where applicable. Please detail housing and husbandry conditions and the source of animals.	NA
9. For experiments involving live vertebrates, include a statement of compliance with ethical regulations and identify the committee(s) approving the experiments.	NA
10. We recommend consulting the ARRIVE guidelines (see link list at top right) (PLoS Biol. 8(6), e1000412, 2010) to ensure that other relevant aspects of animal studies are adequately reported. See author guidelines, under 'Reporting Guidelines'. See also: NIH (see link list at top right) and MRC (see link list at top right) recommendations. Please confirm compliance.	NA

E- Human Subjects

11. Identify the committee(s) approving the study protocol.	NA
12. Include a statement confirming that informed consent was obtained from all subjects and that the experiments conformed to the principles set out in the WMA Declaration of Helsinki and the Department of Health and Human Services Belmont Report.	NA
13. For publication of patient photos, include a statement confirming that consent to publish was obtained.	NA
14. Report any restrictions on the availability (and/or on the use) of human data or samples.	NA
15. Report the clinical trial registration number (at ClinicalTrials.gov or equivalent), where applicable.	NA
16. For phase II and III randomized controlled trials, please refer to the CONSORT flow diagram (see link list at top right) and submit the CONSORT checklist (see link list at top right) with your submission. See author guidelines, under 'Reporting Guidelines'. Please confirm you have submitted this list.	NA
17. For tumor marker prognostic studies, we recommend that you follow the REMARK reporting guidelines (see link list at top right). See author guidelines, under 'Reporting Guidelines'. Please confirm you have followed these guidelines.	NA

F- Data Accessibility

18: Provide a "Data Availability" section at the end of the Materials & Methods, listing the accession codes for data generated in this study and deposited in a public database (e.g. RNA-Seq data: Gene Expression Omnibus GSE39462, Proteomics data: PRIDE PXD000208 etc.) Please refer to our author guidelines for 'Data Deposition'.<br><br>Data deposition in a public repository is mandatory for:<br>a. Protein, DNA and RNA sequences<br>b. Macromolecular structures<br>c. Crystallographic data for small molecules<br>d. Functional genomics data<br>e. Proteomics and molecular interactions	We ensure data are available at www.rcbs.com at https://doi.org/10.2210/pdb7PJE/pdb and https://doi.org/10.2210/pdb7PJF/pdb
19. Deposition is strongly recommended for any datasets that are central and integral to the study; please consider the journal's data policy. If no structured public repository exists for a given data type, we encourage the provision of datasets in the manuscript as a Supplementary Document (see author guidelines under 'Expanded View' or in unstructured repositories such as Dryad (see link list at top right) or Figshare (see link list at top right).	We ensure data are available
20. Access to human clinical and genomic datasets should be provided with as few restrictions as possible while respecting ethical obligations to the patients and relevant medical and legal issues. If practically possible and compatible with the individual consent agreement used in the study, such data should be deposited in one of the major public access-controlled repositories such as dbGAP (see link list at top right) or EGA (see link list at top right).	NA
21. Computational models that are central and integral to a study should be shared without restrictions and provided in a machine-readable form. The relevant accession numbers or links should be provided. When possible, standardized format (SBML, CellML) should be used instead of scripts (e.g. MATLAB). Authors are strongly encouraged to follow the MIRIAM guidelines (see link list at top right) and deposit their model in a public database such as Biomodels (see link list at top right) or JWS Online (see link list at top right). If computer source code is provided with the paper, it should be deposited in a public repository or included in supplementary information.	NA

G- Dual use research of concern

22. Could your study fall under dual use research restrictions? Please check biosecurity documents (see link list at top right) and list of select agents and toxins (APHIS/CDC) (see link list at top right). According to our biosecurity guidelines, provide a statement only if it could.	NA
